# Multi-Omics Analysis Identified Drug Repurposing Targets for Chronic Obstructive Pulmonary Disease

**DOI:** 10.3390/ijms252011106

**Published:** 2024-10-16

**Authors:** Fang Wang, Carlos A. Barrero

**Affiliations:** Department of Pharmaceutical Sciences, Temple University School of Pharmacy, Philadelphia, PA 19140, USA; fang.wang@temple.edu

**Keywords:** chronic obstructive pulmonary disease, bioinformatics, multi-omics data integration, systems biology, drug repurposing

## Abstract

Despite recent advances in chronic obstructive pulmonary disease (COPD) research, few studies have identified the potential therapeutic targets systematically by integrating multiple-omics datasets. This project aimed to develop a systems biology pipeline to identify biologically relevant genes and potential therapeutic targets that could be exploited to discover novel COPD treatments via drug repurposing or *de novo* drug discovery. A computational method was implemented by integrating multi-omics COPD data from unpaired human samples of more than half a million subjects. The outcomes from genome, transcriptome, proteome, and metabolome COPD studies were included, followed by an *in silico* interactome and drug-target information analysis. The potential candidate genes were ranked by a distance-based network computational model. Ninety-two genes were identified as COPD signature genes based on their overall proximity to signature genes on all omics levels. They are genes encoding proteins involved in extracellular matrix structural constituent, collagen binding, protease binding, actin-binding proteins, and other functions. Among them, 70 signature genes were determined to be druggable targets. The *in silico* validation identified that the knockout or over-expression of *SPP1*, *APOA1*, *CTSD*, *TIMP1*, *RXFP1*, and *SMAD3* genes may drive the cell transcriptomics to a status similar to or contrasting with COPD. While some genes identified in our pipeline have been previously associated with COPD pathology, others represent possible new targets for COPD therapy development. In conclusion, we have identified promising therapeutic targets for COPD. This hypothesis-generating pipeline was supported by unbiased information from available omics datasets and took into consideration disease relevance and development feasibility.

## 1. Introduction

Chronic obstructive pulmonary disease (COPD) is currently the third leading cause of death worldwide and sixth in the United States [1,2]. It is characterized by increased breathlessness due to obstructed airflow caused by abnormalities of the airways and/or alveoli [3]. These phenotypes are associated with a long-term dysregulated inflammatory response in the airways and the lungs [4].

Drug discovery and development are increasingly costly, requiring approximately $2.6 billion and 6–15 years for a new FDA-approved medication [5,6]. Repurposing approved therapeutic compounds for new indications can significantly reduce costs and time. Examples include thalidomide’s transformation from a birth-defect-causing drug to an FDA-approved multiple myeloma treatment in 2006 [7], and Pfizer’s repurposing of sildenafil from a cardiovascular drug to Viagra for erectile dysfunction [8].

Multiple strategies have been utilized to identify drug-repurposing opportunities in COPD. One approach is to generate testable drug-repurposing hypotheses based on previous knowledge and expert insights. The bisphosphonate alendronate, a drug indicated for the treatment of osteoporosis, has been reported to induce apoptosis in macrophages [9]. Based on this premise, alendronate inhalation was studied in a mouse model of emphysema to determine its therapeutic utility in COPD. One mouse study demonstrated that alendronate induces apoptosis in alveolar macrophages and inhibits the airspace enlargement, which characterizes COPD [10]. In addition, the high-throughput screening of compound libraries can identify potential lead compounds for targets already linked to COPD. WNT/β-catenin signaling, for example, is known to decrease in patients with COPD. Several compounds that activate WNT/β-catenin signaling *in vitro* and induce lung repair in a mouse model of emphysema have been identified by high-throughput drug screening [11]. Last, genomics-based strategies have also identified novel therapeutic targets for COPD. In a recent genome-wide association study (GWAS) with 588,452 subjects, 1020 signals were identified to be associated with lung function, implicating 559 putative genes; 55 of these genes, including *ITGA2*, have been targeted by drugs in approved or ongoing clinical trials [12]. These example studies highlight that drug-repurposing hypotheses may be helpful in the quest for novel COPD therapies.

The strategies mentioned above were based on a single source of omics data. We propose a multiple-omics integration strategy to help identify novel therapeutic targets for COPD. This project is the first to integrate genome, transcriptome, proteome, and metabolome data from COPD patients’ lungs and bronchoalveolar lavage fluid (BALF). In addition, a distance-based computational model was developed to prioritize potential candidate genes by incorporating interactome and drug-target data. This systems biology approach identifies COPD-associated genes through holistic molecular profiling while evaluating the feasibility of these genes as potential therapeutic targets for COPD. Some of the results of this study have been previously published as an abstract [13].

## 2. Results

### 2.1. Differentially Expressed Genes across COPD Stages

Differential gene expression analyses were performed on the transcriptome data from healthy control subjects and compared against data from ex-smoker patients with mild (GOLD 1), moderate (GOLD 2), severe (GOLD 3), and very severe (GOLD 4) COPD to explore the dysregulated genes across different stages of COPD. The dataset GSE47460 from the Gene Expression Omnibus database was used as the discovery set of 203 COPD patients. Patient demographic information is recorded in Table 1, which includes 23 GOLD 1, 94 GOLD 2, 32 GOLD 3, and 54 GOLD 4 patients.

A comparison of the transcriptomic data between the control and early-stage COPD patients identified five differentially expressed genes (DEGs) in the GOLD 1 group and nine DEGs (three up-regulated and six down-regulated genes) in the GOLD 2 group. In contrast, 83 DEGs (67 up-regulated and 16 down-regulated genes) were identified between the control and GOLD 3 group, and 168 DEGs (125 up-regulated and 43 down-regulated genes) were identified between the control and GOLD 4 group (adjusted *p* < 0.05 and fold change > 2 or <0.5, Table S1).

**Figure 1:**
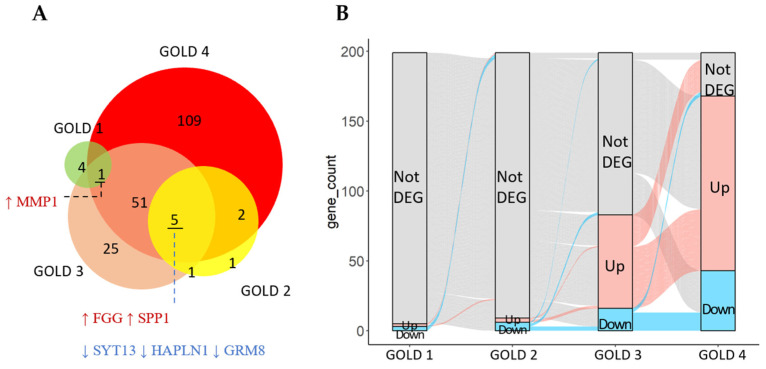
(**A**) Genes consistently up-regulated (gene name in red) or down-regulated (gene name in blue) across COPD stages. Each circle represents the number of DEGs identified in individual GOLD levels. (**B**) The numbers of DEGs identified across different stages of COPD. The flows between each GOLD column represent the status change of genes in the down-regulated (blue), up-regulated (red), and not differentially expressed (grey) categories. DEG: Differentially expressed genes.

The overlap between the DEGs identified across the different stages of COPD is shown in Figure 1A. Several genes were consistently up-regulated or down-regulated across COPD stages. These include *MMP1*, *FGG*, *SPP1*, *SYT13*, *HAPLN1*, and *GRM8*, each demonstrating an altered expression starting at the early COPD stages of GOLD 1 and 2. Some genes were only dysregulated in the later stages of COPD (Figure 1B).

### 2.2. Differentially Expressed Genes in GOLD 4 COPD

**Figure 2:**
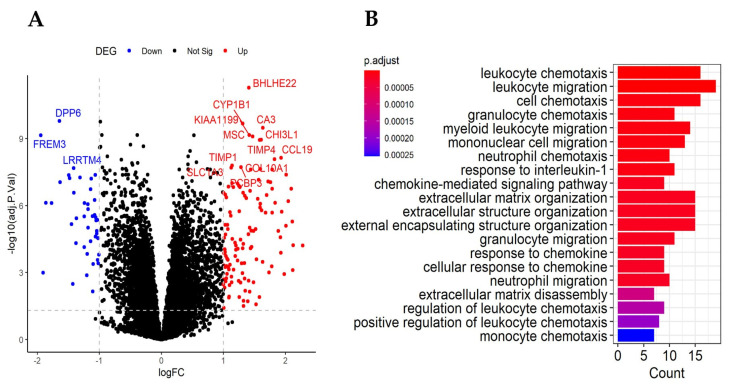
(**A**) DEGs between healthy subjects and GOLD 4 COPD patients. Dotted lines represent the DEG cutoffs of adjusted *p* < 0.05 and |log2 fold change| ≥ 1. Significantly up-regulated and down-regulated genes are shown as red and blue dots. (**B**) Top 20 over-represented biological process pathways in GOLD 4 patients. Color indicates the adjusted *p* from the GO enrichment analysis, and *x*-axis represents the count of the measured genes in the pathway.

Due to the heterogeneity of COPD, we focused our subsequent analyses on the most severe COPD subjects, namely, the GOLD 4 patients. In the discovery set, the transcriptomic analysis identified 168 DEGs between GOLD 4 patients and healthy control subjects, including 125 up-regulated and 43 down-regulated genes (adjusted *p* < 0.05, |log2 fold change| ≥ 1) (Figure 2A). A gene ontology (GO) enrichment analysis on DEGs revealed 161 statistically significant over-represented biological processes, highlighting immune response events in GOLD 4 patients compared with healthy control patients (adjusted *p* < 0.05). These pathways included leukocyte chemotaxis, granulocyte chemotaxis, and extracellular matrix organization (Figure 2B). Compared with a similar GOLD 4 validation dataset (GSE76925), the 168 DEGs were supported by both gene- and pathway-level validation approaches (see the Online Supplement).

### 2.3. Signature Genes from Genomics, Proteomics, and Metabolomics Analysis

**Figure 3:**
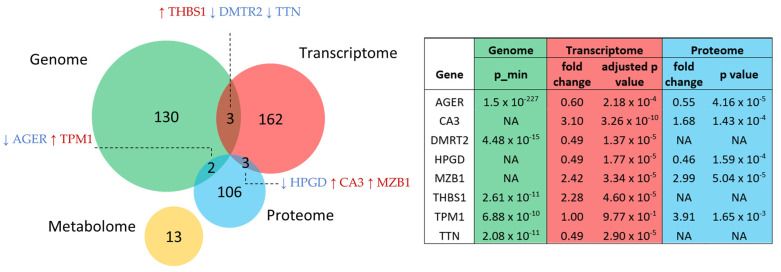
Venn diagram of the number of signature genes at each omics level, with overlapping signature genes and their corresponding *p* and fold change. The names of the overlapping up-regulated and down-regulated genes are shown in red and blue, respectively. *p*_min: The *p* of the most significant variant implicating the gene is reported in the table based on its association with the lung function. In the proteomics data, nominal *p* are provided due to the lack of raw data.

Genomics, proteomics, and metabolomics data from COPD patients were included in the pipeline to complement the transcriptomic signatures (Table S2). In a recent multi-ancestry genome-wide association meta-analysis, 1020 independent signals were found to be associated with lung functions, implicating 135 genes supported by at least two variant-to-gene mapping evidences (Table S3). Five COPD lung proteomics studies were analyzed, and 113 proteins were identified as differentially expressed in COPD patients compared with healthy control subjects (Table S4). The metabolomics data from BALF were used to identify additional lung proteins potentially associated with COPD through the Metabolite Annotation and Gene Integration (MAGI) algorithm. One BALF metabolomics study identifying 25 metabolites associated with COPD was also included in our pipeline (Table S5). The MAGI algorithm searched its biochemical reaction database and identified 33 unique proteins highly associated with the 25 metabolites (confidence score > 4). The 13 genes corresponding to the 33 proteins were included in the pipeline for integration. *AGER*, *TPM1*, *THBS1*, *DMTR2*, *TTN*, *HPGD*, *CA3*, and *MZB1* were identified as signature genes on more than one omics level (Figure 3, Table S6).

In summary, we identified 429 signature genes from the re-analysis of the multiple-omics data, including 135 from the genome, 168 from the transcriptome, 113 from the proteome, and 13 from the metabolome (Figure 3). Eight genes were identified by more than one omics level.

### 2.4. COPD Signature Genes by Interactome Distance Network

To increase the overlap between the signature genes identified on each omics level, the selection of candidate genes was expanded by including genes directly interacting with these signature genes using the STRING database. Then, 11,759,455 protein–protein interactions of physical and functional associations were retrieved from the STRING database for 19,344 genes in the database. High-confidence (STRING score > 0.7) protein–protein interactions of physical and functional associations were retrieved to minimize the likelihood of computational artifacts and random association. It was found that 841,069 (7.15%) of the association had a score greater than 0.7. These interactions were included in the analysis. Subsequently, a distance-based network algorithm was created to rank the candidate genes based on their closeness to signature genes identified at all omics levels. We aim to prioritize candidate genes that are signature genes from one omics level, and also interact with signature genes/proteins identified from other omics levels.

**Figure 4:**
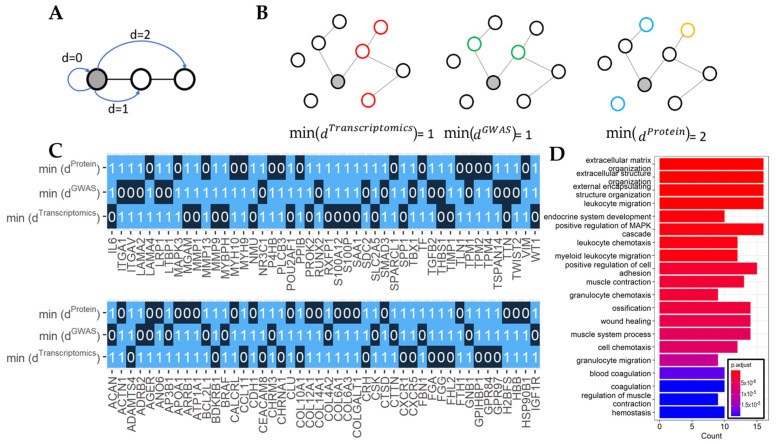
(**A**) Illustration of distances between genes in the network. The grey circle is the candidate gene, and the white circles are the additional genes in the network. Black lines represent physical or functional interactions between the genes. Blue lines were added to denote candidate genes’ distances d from the network’s genes. (**B**) Shortest distances of a candidate gene from all omics signature genes in the protein-protein interaction network. Interacting proteins are linked with black lines. The shortest distances of a candidate gene (gray circle) to signature genes from the transcriptomics (red circles), genomics (green circles), and proteomics signature genes (blue circles) and proteins linked to COPD-associated metabolomics (yellow circles) are 1, 1, and 2 respectively. (**C**) Ninety-two prioritized candidate genes with close proximity to all omics levels. Numbers represent the shortest distance of the candidate gene from the omics level on each row. (**D**) Top 20 significantly enriched pathways for the 92 COPD signature genes. Color indicates the adjusted *p* from the GO enrichment analysis, and *x*-axis represents the count of the measured genes in the pathway.

The distance network evaluated such a relationship by calculating the interaction distance between the candidate genes and signature genes from transcriptomics, genetic, and protein level (Equation (1)). For each gene, the sum of its shortest distance to three sets of signature genes was calculated to represent the overall proximity of a candidate gene to the signature genes identified at different omics levels (Figure 4A,B). A total of 6917 direct neighbors to the signature genes were identified. Among this expanded list of genes, 92 were prioritized as the final set of COPD candidate signature genes. The 92 genes are selected based on two types of interaction distances (Figure 4C). A distance of 0 indicates the specific gene is identified as the signature gene on the corresponding omics level. A distance of 1 indicates the specific gene directly interacts with signature genes from the other omics levels. Thus, the 92 genes represent genes in close proximity to the signature genes identified at different omics levels—all of them were identified on one omics level and were also direct neighbors to signature genes on other omics levels. They are composed of genes involved in the extracellular matrix structural constituent (*ACAN*, *COL12A1*, *COL6A3*, *LAMA2*, *COL10A1*, *FBN1*, *FGG*, *LAMA4*, *FGA*, and *COL4A2*), cell chemotaxis (*ANO6*, *CCL11*, *CXCR1*, *CXCR5*, *IL6*, *ITGA1*, *MAPK3*, *PPIB*, *S100A12*, *SAA1*, *TGFB2*, and *THBS1*), the epithelial cell apoptotic process (*ANO6*, *BCL2L1*, *BRAF*, *FGA*, *FGG*, *IGF1R*, *IL6*, and *THBS1*), the response to transforming growth factor beta (*COL4A2*, *FBN1*, *IGF1R*, *LTBP1*, *NR3C1*, *SMAD3*, *TGFB2*, and *THBS1*), coagulation (*ANO6*, *AP3B1*, *FGA*, *FGG*, *HBB*, *IL6*, *MYH9*, *SAA1*, *THBS1*, and *TLN1*), and genes with other functions (Figure 4D, Table S7).

*ACAN* is one of the prioritized potential drug targets identified by our pipeline. This gene was not only genetically associated with COPD but was also directly linked to COPD signature genes identified on the transcriptomics and proteomics levels.

### 2.5. Druggable Targets and In Silico Validation

**Figure 5:**
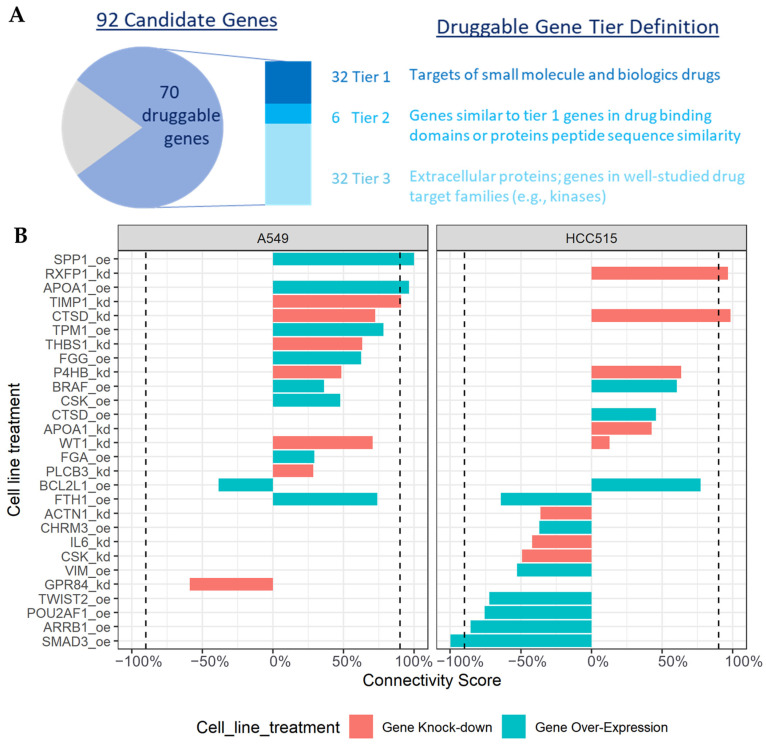
(**A**) Druggable gene targets (blue section of the pie chart) among the 92 COPD signature genes and their druggable gene tiers. The grey section of the pie chart represents genes that are not classified as druggable or have unknown druggability. (**B**) COPD signature genes with high connectivity scores through *in silico* perturbation evaluation based on gene knock-down (red bar) or gene over-expression (green bar) treatment in cell line experiments. Dashed lines represent high connectivity score of 90% or −90%. A549 is a cell line of human adenocarcinoma alveolar basal epithelial cells. HCC515 is a cell line of human non-small-cell lung adenocarcinoma.

Of the 92 COPD signature genes, 70 were considered druggable targets based on the definition from the druggable genome study [14] (Figure 5). These druggable genes are targets of existing drugs, genes with protein structures or sequences similar to these targets, or genes in well-studied drug target families, such as the kinase family of enzymes. Among these 70 druggable genes, 32 demonstrated Tier 1 druggability, indicating they were targets of approved drugs or drug candidates in clinical trials. Therefore, they could be considered prioritized targets for potential COPD treatment. By extracting drug–gene target pair information from the DrugBank database, we identified 393 drugs or biomedical entities targeting the 32 druggable genes in Tier 1 (Table S8), including spermine, andrographolide, and several drugs currently prescribed for the management of COPD symptoms. Interestingly, of the 32 drugs reported in DrugBank for the treatment of COPD, 21 were identified by our pipeline.

In addition, we performed an *in silico* evaluation of the potential effect of the genetic modifications of the 92 candidate genes with the Connectivity Map (CMap) database as an alternative validation step. This database provides transcriptomic profiles of human cell lines that underwent knockout or over-expression experiments for 3848 unique genes, covering 41 of the 92 COPD signature candidate genes. Through the CMap analysis, the knockout or over-expression of six COPD candidate genes were found to result in transcriptomic profiles positively or negatively associated with COPD (Figure 5B). The over-expression of *SPP1* and *APOA1* genes and the knockout of the *CTSD*, *TIMP1*, and *RXFP1* genes were positively connected with COPD transcriptomics (connectivity scores > 90%), indicating a transcriptomic pattern similar to COPD. Conversely, the over-expression of *SMAD3* genes was observed to be negatively connected with the COPD transcriptomics profile (connectivity scores < –90%), indicating a reversed transcriptomic profile compared with COPD. Therefore, we hypothesize that the inhibitors or antagonists targeting the *SMAD3* gene may be further evaluated as COPD therapies as they might lead to a reversed transcriptomic change pattern compared with COPD.

In summary, we have used an *in silico* validation analysis to corroborate the involvement of *SPP1*, *APOA1*, *CTSD*, *TIMP1*, *RXFP1*, and *SMAD3* as causal or upstream genes that may drive the cell transcriptomics to a status similar to or contrasting with COPD, suggesting their potential as therapeutic targets for intervention.

## 3. Discussion

Our work is the first study that evaluates both the disease association and the target development feasibility of COPD candidate genes through the systematic integration of four types of omics data. There have been other COPD omics studies, but they primarily relied on one or two types of omics data and aimed to explain the pathogenesis or subtypes of COPD [13,15,16,17]. Moreover, our study is particular in selecting omics data from lung tissue and BALF samples to ensure a more direct representation of COPD-involved organs, as opposed to blood or plasma in previous studies.

We first identified DEGs across different stages of COPD. The number of DEGs increased as COPD progressed to later stages. Considering disease heterogeneity and the lower detection power of signature genes in the early stages, our current pipeline focused on the DEGs in GOLD 4 patients. The transcriptomic analysis revealed 43 down-regulated and 125 up-regulated genes in GOLD 4 COPD patients. The higher count of up-regulated genes aligns with prior findings that smoking leads to chromosome opening and increased active transcription [18]. The disrupted immune response and altered extracellular composition are the hallmarks of COPD [19,20]. Related pathways were observed to be over-represented in the functional analysis of these DEGs. The 168 DEGs were validated by both the gene set and pathway approaches in comparison with a similar COPD dataset. Although the DEGs exhibited limited overlap in the discovery and validation sets, this does not negate the validity of most identified DEGs, illustrated in a microarray reproducibility study [21].

Genomics, proteomics, and metabolomics data were also included in our pipeline. Genomics data can provide signatures from the genome-wide level over a vast population. Proteomics and metabolomics data, although with a lower throughput, can provide signatures related to the downstream functional molecules closely related to the disease status. However, we observed a minimum overlap between the signatures from these omics levels. This was also observed in another study of the genomics and transcriptomics integration in COPD [22]. For example, we identified matrix metalloproteinase 13 as a protein associated with COPD in proteomics data but not in transcriptomic data. Previous studies indicated that protein abundance does not always correlate well with mRNA expression [23]. Therefore, incorporating various omics data is essential for a comprehensive understanding of the biological processes across multiple molecular levels.

Due to the limited number of overlapping genes identified between different omics technologies, we integrated them with the interactome data to expand the candidate gene pool and enable prioritization using distance matrices. Our distance-based network model prioritized 92 genes as the final set of COPD candidate genes due to their overall proximity to all omics levels. A pathway analysis of the 92 signature genes highlighted disease-development-associated pathways that could serve as potential pharmacological targets for COPD treatment, including epithelial cell apoptosis, cell chemotaxis, the response to transforming growth factor-beta, and coagulation. We compared our distance model with a heat diffusion network model and observed minimal overlap due to their distinct underlying assumptions. The heat diffusion model prioritizes genes with fewer neighbors [24], which could introduce bias due to the incomplete interactome data. Conversely, our model prioritizes genes connected with many neighbors. Although well-studied genes could be favored in such cases, the supporting protein–protein interactions have a high confidence score to ensure a robust connection to candidate genes.

We further evaluated the target development feasibility regarding their drug-repurposing potential and *in silico* target perturbation evaluation. Interestingly, 70 of 92 candidate genes were considered druggable, while only 22% of the genes in the human genome are considered druggable [14]. Since COPD is a complex disease involving multiple pathways and genes, it is not surprising to find COPD-related genes targeted by drugs that treat other conditions. Thirty-two candidate genes were Tier 1 druggable genes, which are the targets of approved drugs or drug candidates in clinical trials. We identified 393 drugs or biomedical entities targeting these 32 genes. One of the examples is spermine, a potent regulator of inflammatory responses [25]. In the recent studies, spermine demonstrated a protective role in the lung and myogenesis in COPD [26,27]. Another example is andrographolide, a natural anti-inflammatory agent. It has been reported to show antioxidative benefits against cigarette-smoke-induced lung injury in a mouse *in vivo* model [28]. Interestingly, our pipeline also identified 21 current drugs used for the treatment of COPD, including Tiotropium, Formoterol, Salmeterol, and others. These examples support the theory that a drug-repurposing strategy may offer new treatment opportunities for a complex disease like COPD. Other drugs have not been explored for their role in COPD, but several of them are used for treating immune and nervous system diseases or cardiovascular disorders, suggesting common pathways are impacted in COPD as well [29].

Conventionally, only a handful of candidate genes will be empirically chosen for validation by an *in vitro* or *in vivo* experiment. In contrast, we performed an *in silico* evaluation and identified six COPD candidate genes that may lead to transcriptomic profiles positively or negatively associated with COPD. These genes are likely the upstream genes contributing to COPD patients’ dysregulated gene expression profiles. *SPP1*, the gene encoding secreted phosphoprotein 1 or osteopontin, is one of the *in silico* validated genes. The over-expression of *SPP1* leads to a transcriptomic profile similar to COPD. Moreover, we identified it as one of the few genes dysregulated across COPD stages. *SPP1* was also suggested as a potential biomarker for COPD exacerbation [30]. In the phases I and II clinical trials for rheumatoid arthritis, the drug ASK8007, a monoclonal antibody targeting *SPP1*, was investigated and presented no safety concern [31].

*SMAD3* is another *in silico* validated gene identified by our pipeline as a potential drug-repurposing target. In our pipeline, *SMAD3* showed a genetic association with COPD and directly interacted with signature genes identified from the transcriptomic and protein level. In the cMap analysis, the over-expression of *SMAD3* genes was observed to be negatively connected with the COPD transcriptomics profile. Previously, the Smad3 signaling pathway has been shown to be involved in emphysema [32]. One study has found that microRNA-145 targets *SMAD3* and negatively regulates pro-inflammatory cytokine release in COPD [33]. Another study showed that ligustilide, a novel Smad3 covalent inhibitor, successfully suppressed airway remodeling in the COPD mice model [34]. The above findings indicate the high potential of *SPP1* and *SMAD3* as drug-repurposing targets for COPD and the success of our pipeline in discovering such a target.

Certain limitations exist within this study. First, the current study solely focused on the ex-smokers to control for the impact of smoking due to the limited number of smokers in the dataset. Nonetheless, a considerable percentage of COPD patients persist in smoking despite their diagnosis [35]. Future research with a sufficient number of lung samples from smokers with COPD will enable the identification of signatures that are more pertinent to this population. Second, the omics data used in this project did not include information on exacerbations. COPD exacerbations have been shown to alter molecular profiles [36]. While plasma-based omics data with exacerbation information are available, our study focuses on omics data from lung tissue. Our pipeline could be instrumental in future studies by incorporating exacerbations as a phenotype for omics data analysis. Third, protein detection coverage is limited in the published proteomics studies, with a throughput of up to thousands of proteins. We compensated for this by identifying additional proteins linked to COPD-associated metabolites. However, the metabolomics data themselves are subject to low detection coverage, as well as the more significant challenge of assigning the metabolite structure to the metabolomics feature [37]. Fourth, the cell type specificity was not taken into consideration in the current pipeline. This can be a future direction of investigation when there are more publicly available omics data generated by single-cell RNA-Seq or data from specific cell types in the lung. In addition, the cell lines selected for the CMap evaluation consist of two lung adenocarcinoma cell lines. Although we have chosen the two cell lines most representative of the lung tissue from the database, future *in vitro* experiments with non-cancer cell lines will be more relevant to COPD. Lastly, inter-individual variability was not adjusted in our pipeline due to the lack of omics measurements from paired samples. There is one 2023 paper from Zhang et al. which measured paired samples for multi-omics integration [38], but such data are currently still very limited. Therefore, at this stage, we focus first on integrating the summary level omics data to generate new insights on the COPD population, especially with the increased availability of the individual omics-level data across studies.

This study represents the first systematic approach integrating four types of omics data to explore drug-repurposing targets for COPD. Seventy genes were identified by our pipeline as candidate drug-repurposing targets based on their overall proximity to COPD signature genes and their druggable properties. The hypothesis-generating pipeline is supported by rich information from omics data, not limited by our prior knowledge; it evaluates both the disease relevance and development feasibility of targets and can be readily applied to other diseases. Future directions will include expanding the pipeline to the earlier stage of COPD, capturing network relationships across omics levels with advanced computation models, and the *in vitro* and *in vivo* validation of the prioritized candidate genes with lung cell lines and tissues.

## 4. Materials and Methods

### 4.1. Study Population and Omics Data

Gene expression data were obtained from lung samples of 268 COPD GOLD 1-4 patients and control subjects [39]. Gene expression data from a similar COPD cohort (*n* = 150) were used as the validation set for COPD transcriptomic signatures [22]. Genomic data were curated from a recent GWAS meta-analysis of lung functions in 588,452 subjects from 49 cohorts [12]. Proteomic data of lung tissue samples (*n* = 155) of COPD patients and controls were obtained from five previous studies [38,40,41,42,43]. Metabolomic data from BALF samples of 115 COPD patients and healthy control subjects were also included [44]. Comprehensive information about each omics dataset used for this analysis is available in the Online Supplementary Data.

### 4.2. Transcriptomic Analysis

Differential gene expression analyses were performed using the *limma* package (v3.36.5) in R [45]. Healthy controls were compared with subjects in each COPD stage as defined by the Global Initiative for Chronic Obstructive Lung Disease (GOLD) criteria. Functional enrichment analysis of the DEGs was conducted through the GO enrichment analysis method with the clusterProfiler package in R [46,47]. The DEGs identified in the discovery set were confirmed in the validation set using hypergeometric and gene set enrichment tests [48,49].

### 4.3. Metabolomics, Proteomics, and Genomics Signatures

Genomic, proteomic, and metabolomic signatures in COPD patients’ lungs and BALF were identified through a literature search. The MAGI software v1.0 was used to identify proteins and reactions linked to the metabolites associated with COPD [50]. Proteins work as enzymes that catalyze the biological reactions to produce metabolites. Our assumption is that, if a dysregulated metabolite is associated with a certain phenotype or disease, the protein involved in the reaction to generate the specific metabolite are also dysregulated. MAGI searches its biochemical reaction database with sources from MetaCyc and RHEA to identify the proteins involved in the reactions for the metabolites of interest. Protein signatures were mapped to gene names for pipeline integration. Genetic loci and implicated genes were obtained from the original GWAS paper, where SNPs were mapped using a variety of approaches including genetic region annotation, linkage disequilibrium, eQTL, variant functional consequences, ancestry analysis, or a combination of these approaches. The common genes between gene signatures from each omics level were identified using a Venn diagram.

### 4.4. Omics Integration

We included the interactome data in the STRING database v11 [51] to build the systems biology pipeline for integrative analysis with our previously identified signatures. STRING database provides the physical and functional protein–protein interaction data based on several types of sources, including experimental data, computational prediction, and public literature text mining. We filtered for interactions with high-confidence-level interactions (interaction score ≥ 0.7) to be included in the analysis to minimize the likelihood of computational artifacts and random association. A distance-based network model was developed to integrate omics and rank candidate genes on their proximity to signature genes on all omics levels (Equation (1)). The model was implemented in R software (v4.2.2). See Supplementary Data for detailed R running environment and code availability.

(1) $${\hat{d}}_{i}^{sum}=\underset{j}{\min}d_{ij}^{Transcriptomics}+\underset{j}{\min}d_{ij}^{GWAS}+\underset{j}{\min}d_{ij}^{Protein}$$

Equation (1): For each *i*, we calculated its distance to each signature gene *j* from the transcriptomics level, and then obtained the minimal distance, denoted as min *d_ij_*, among all the gene *j*. Similarly, we obtained the minimal distances to genomic signature genes and protein signatures from the proteomics and metabolomics levels. Finally, we summed the three minimal distances to create a total distance, representing the overall proximity of candidate gene *i* to all omics signature genes.

### 4.5. Drug Repurposing and In Silico Validation

The final steps in the pipeline were druggability analysis and *in silico* validation. The druggability of a candidate gene was assessed based on the definition from the previous druggable genome study [14]. These genes were categorized into three tiers based on their use in approved therapeutics or ongoing clinical trials, protein similarity to the drug targets of approved therapeutics, and protein classes (e.g., well-studied drug target families). Drugs targeting the druggable genes were retrieved and filtered from the DrugBank database (version 5.1.4). The CMap database (L1000) was used for *in silico* validation to evaluyate the effect of knockout or over-expressing of candidate genes in human cell lines [52].

## 5. Conclusions

Through the integration of genomic, lung transcriptomic and proteomic, BALF metabolomic, and interactome data, we have uncovered 92 genes as COPD signatures, and 70 of them were determined to be druggable targets. Further *in silico* validation highlighted the *SPP1*, *APOA1*, *CTSD*, *TIMP1*, *RXFP1*, and *SMAD3* genes as promising candidates for therapeutic intervention.

## 6. Patents

This work has a provisional patent application: Methods for multi-omics analysis drug repurposing identification for Chronic Obstructive Pulmonary Disease. Invention Disclosures: TTD2022-092, C2024-052. Provisional Application: 63/702,292 10/02/2024.

## Figures and Tables

**Table 1:** Basic demographics of the subjects in the discovery set grouped by GOLD criteria.

	Healthy (0)	GOLD 1 (1)	GOLD 2 (2)	GOLD 3 (3)	GOLD 4 (4)	*p*	Significant Intergroup Differences *
Totals	65	23	94	32	54	NA	NA
Age, yr, mean ± SD	65.6 ± 10.4	70.9 ± 8.4	67.8 ± 8.9	65.5 ± 8.2	57.2 ± 8.4	<0.0001	(0) > (4) (1) > (4) (2) > (4) (3) > (4)
Sex, M/F	36/29	17/6	57/37	19/13	24/30	0.15	No intergroup difference
Smoking status, Current/ Ever/Never	2/63/0	0/21/2	11/79/4	3/28/1	0/53/1	0.0107	No intergroup difference

* All significant intergroup differences listed achieved a Bonferroni-corrected *p* of *p* < 0.005.

## Data Availability

The code and the raw data supporting the conclusions of this article will be made available by the authors upon request.

## Supplementary Materials

The following supporting information can be downloaded at: https://www.mdpi.com/article/10.3390/ijms252011106/s1. References [53,54,55,56,57,58,59,60,61,62,63,64,65] are cited in the supplementary materials.
