# Supplementary material for: Multi-Omics Analysis Identified Drug Repurposing Targets for Chronic Obstructive Pulmonary Disease
Source: Int J Mol Sci. 2024 Oct 16;25(20):11106. doi: 10.3390/ijms252011106 (PMC11507528; doi:10.3390/ijms252011106)
Supplement: Supplementary file 1 [file ijms-25-11106-s001.zip › ijms-3236357-supplementary.pdf]

Multi-Omics Analysis Identified Drug Repurposing Targets for Chronic Obstructive Pulmonary Disease

Authors: Fang Wang and Carlos Barrero

Online Data Supplement

TABLE OF CONTENTS

Page

1 Supplement Figures and Tables 4

Table S5. Differentially expressed genes (DEG) in GOLD I to IV patients vs healthy controls 4

Table S6. Sources for omics data 11

Table S7. Summary of the variant-to-gene mapping evidence for the 135 suspected genes associated with COPD 12

Table S8. Proteomics studies on lung tissues from COPD patients 13

Table S9. Metabolites input for MAGI 14

Table S10. Signature genes identified on more than one omics levels 16

Table S11. Significantly enriched pathways for the 92 COPD signature genes 17

Table S12. Number of drugs targeting the tier 1 druggable genes 27

2 Supplement Result 30

2.1 Validation of GOLD IV DEGs 30

3 Material and Method 38

3.1 Transcriptomic data 38

3.2 Differential expression analysis 39

3.3 Functional enrichment.. 39

3.4 Gene set enrichment analysis 40

3.5 Over-representation test of the differentially expressed genes 40

3.6 GWAS data 42

3.7 Proteomics data 42

3.8 Metabolomics data 43

3.9 Integration of metabolomics data with MAGI 43

3.10 Protein-protein interaction data.. 43

3.11 Calculation of distances between candidate genes and omics signature genes.. 44

3.12 Heat diffusion network analysis with Cytoscape 46

3.13 The retrieval of drug-gene target information... 46

3.14 The automated literature search for COPD studies with repurposing drugs...... 47

3.15 Connectivity map analysis 47

3.16 R for data analysis.. 48

4 References (Supplement Only).. 51

1 Supplement Figures and Tables

Table S1. Differentially expressed genes (DEG) in GOLD I to IV patients vs healthy controls

logFC	AveExpr	P.Value	adj.P.Val	probe	group	GENE_SYMBOL
-1.18635	10.68327	8.17E-08	0.000416	A_24_P224116	GOLD I	PLA2G1B
-1.01932	10.43221	5.15E-06	0.00414	A_32_P142440	GOLD I	PCSK9
-1.28458	11.78332	1.29E-05	0.00647	A_23_P7965	GOLD I	PGC
1.059249	8.544712	6.14E-05	0.015888	A_24_P161144	GOLD I	ZNF843
1.929994	7.724092	0.000103	0.023394	A_23_P1691	GOLD I	MMP1
-1.16848	5.73862	5.39E-06	0.001613	A_32_P8221	GOLD II	GRM8
-1.25223	5.312968	2.04E-05	0.00352	A_23_P7727	GOLD II	HAPLN1
1.119894	6.153789	3.13E-05	0.004366	A_23_P101683	GOLD II	CLC
-1.17008	4.826441	5.90E-05	0.005668	A_23_P94517	GOLD II	DBC1
-1.0728	4.010685	6.60E-05	0.005836	A_23_P161659	GOLD II	SYT13
-1.50556	4.434363	0.000287	0.013211	A_32_P780817	GOLD II	CT45A1
1.037109	9.139393	0.000815	0.024059	A_23_P7313	GOLD II	SPP1
1.20459	11.64304	0.001456	0.032924	A_23_P148088	GOLD II	FGG
-1.08113	5.569232	0.001687	0.035608	A_32_P170547	GOLD II	CT45A5
-1.40467	6.728884	5.20E-11	7.21E-07	A_23_P17826	GOLD III	SLC5A1
-1.5293	6.625044	1.58E-10	7.21E-07	A_23_P170888	GOLD III	DPP6
1.560738	8.904054	1.89E-10	7.21E-07	A_23_P20316	GOLD III	CA3
1.138171	9.095764	1.80E-09	3.44E-06	A_32_P75581	GOLD III	BHLHE22
1.555577	7.537411	3.82E-09	6.49E-06	A_23_P253321	GOLD III	PNOC
2.046599	5.786577	4.55E-09	6.95E-06	A_24_P95723	GOLD III	KIAA0125
1.704752	5.438078	2.43E-08	2.41E-05	A_24_P252945	GOLD III	CXCR5
2.153289	5.403305	2.52E-08	2.41E-05	A_23_P46039	GOLD III	FCRLA
-1.11594	6.430881	5.53E-08	3.95E-05	A_24_P68183	GOLD III	EGFEM1P
1.628398	10.6328	5.69E-08	3.95E-05	A_23_P84596	GOLD III	MZB1
-1.54852	8.64565	7.08E-08	4.32E-05	A_23_P65307	GOLD III	SLITRK6
-1.8439	5.73862	1.05E-07	5.51E-05	A_32_P8221	GOLD III	GRM8
-1.55121	5.023062	1.60E-07	6.97E-05	A_24_P738168	GOLD III	FREM3
1.10837	8.118916	2.30E-07	8.34E-05	A_23_P324754	GOLD III	KIAA1199
1.623874	8.330826	2.52E-07	8.63E-05	A_23_P113572	GOLD III	CD19
1.156641	4.932203	2.66E-07	8.63E-05	A_23_P256948	GOLD III	MSC
1.570442	6.506955	3.19E-07	9.48E-05	A_23_P31725	GOLD III	BLK
1.52114	11.06426	3.29E-07	9.48E-05	A_23_P312920	GOLD III	POU2AF1
1.070631	6.307773	3.82E-07	0.000108	A_23_P207201	GOLD III	CD79B

logFC	AveExpr	P.Value	adj.P.Val	probe	group	GENE_SYMBOL
1.579912	7.835862	4.27E-07	0.000114	A_23_P201211	GOLD III	FCRL5
1.084486	3.858757	4.71E-07	0.00012	A_23_P147255	GOLD III	PCBP3
-1.17057	10.50269	5.03E-07	0.000126	A_23_P113793	GOLD III	ZBED2
1.103152	7.297388	7.74E-07	0.000166	A_23_P153390	GOLD III	CLEC4G
1.623433	3.907201	9.02E-07	0.000186	A_23_P152262	GOLD III	DPEP1
1.129605	6.823864	9.82E-07	0.000199	A_23_P214360	GOLD III	IRF4
1.093864	9.075132	9.93E-07	0.000199	A_23_P48088	GOLD III	CD27
-1.27223	4.263608	1.03E-06	0.000204	A_23_P217277	GOLD III	SLITRK2
-1.51327	4.794553	1.16E-06	0.000219	A_24_P349117	GOLD III	GPR158
1.510528	11.08337	1.39E-06	0.000234	A_23_P123853	GOLD III	CCL19
1.469686	8.571854	1.60E-06	0.00026	A_23_P37736	GOLD III	TNFRSF17
1.215376	6.938401	1.78E-06	0.00028	A_23_P39067	GOLD III	SPIB
1.240004	4.198019	1.84E-06	0.000284	A_23_P159316	GOLD III	BFSP2
1.357625	14.9849	1.95E-06	0.000286	A_23_P120812	GOLD III	IGLL1
-1.09894	5.580312	2.81E-06	0.000358	A_23_P169351	GOLD III	SH3GL2
1.179619	3.382741	3.43E-06	0.000397	A_23_P84705	GOLD III	TNFRSF13B
1.268782	3.711818	3.53E-06	0.000399	A_32_P140489	GOLD III	GDF6
1.271816	7.251063	4.53E-06	0.000487	A_23_P79572	GOLD III	MGC16025
1.786762	3.76122	5.04E-06	0.00052	A_23_P432947	GOLD III	GREM1
-1.63447	4.010685	6.24E-06	0.000599	A_23_P161659	GOLD III	SYT13
-1.16374	9.331937	6.95E-06	0.000636	A_23_P2674	GOLD III	KRT4
1.555548	5.736843	7.53E-06	0.000664	A_23_P13548	GOLD III	CHRDL2
1.987443	7.724092	8.15E-06	0.000698	A_23_P1691	GOLD III	MMP1
1.184429	10.28468	1.03E-05	0.000828	A_23_P145096	GOLD III	PLA2G7
1.192178	6.910125	1.03E-05	0.000828	A_23_P41365	GOLD III	SMR3A
1.006787	8.823965	1.07E-05	0.000832	A_23_P58251	GOLD III	CPZ
1.018526	6.824006	1.35E-05	0.000943	A_23_P16953	GOLD III	HTR2B
1.155203	4.024579	1.43E-05	0.000956	A_23_P16384	GOLD III	NLRP7
1.517676	6.654159	1.88E-05	0.001119	A_23_P165598	GOLD III	DAPL1
1.380472	9.514173	2.20E-05	0.001269	A_24_P264943	GOLD III	COMP
1.01452	3.204875	2.22E-05	0.001271	A_23_P151778	GOLD III	CMA1
1.027442	6.933893	2.40E-05	0.001335	A_23_P131024	GOLD III	ZBTB32
1.321261	6.845509	2.61E-05	0.001418	A_23_P85209	GOLD III	IL13RA2
1.474283	10.40163	3.04E-05	0.001557	A_23_P166408	GOLD III	OSM
1.26413	11.12434	3.17E-05	0.001602	A_23_P140384	GOLD III	CTSG
1.009822	4.174818	5.10E-05	0.002166	A_23_P151637	GOLD III	RNASE2
1.163479	6.017402	5.20E-05	0.00218	A_23_P397320	GOLD III	JSRP1
1.033314	7.077879	6.22E-05	0.002397	A_23_P160159	GOLD III	SLC2A5
1.323711	7.567848	9.37E-05	0.003218	A_23_P77493	GOLD III	TUBB3

logFC	AveExpr	P.Value	adj.P.Val	probe	group	GENE_SYMBOL
-1.51641	5.312968	0.000111	0.003562	A_23_P7727	GOLD III	HAPLN1
1.009437	8.377014	0.000113	0.003611	A_23_P118158	GOLD III	HS3ST2
1.829474	7.152695	0.000114	0.003611	A_23_P321949	GOLD III	PLA2G2A
1.028818	4.366662	0.000116	0.003674	A_23_P250302	GOLD III	CCR3
1.218912	7.958157	0.000117	0.003691	A_23_P2645	GOLD III	SDS
1.149944	4.953527	0.000119	0.003738	A_24_P695306	GOLD III	TMEM229A
2.10116	5.449969	0.000121	0.003768	A_23_P121695	GOLD III	CXCL13
1.136353	8.269318	0.000136	0.004032	A_23_P207456	GOLD III	CCL8
1.347978	6.153789	0.000173	0.004842	A_23_P101683	GOLD III	CLC
1.05625	5.423022	0.000175	0.004865	A_23_P160751	GOLD III	FCRL2
-1.29598	9.244094	0.000302	0.006974	A_23_P145841	GOLD III	SOSTDC1
1.05738	7.366384	0.000302	0.006983	A_23_P51787	GOLD III	AMPD1
1.024834	8.087427	0.000481	0.009158	A_23_P214144	GOLD III	COL10A1
1.135181	6.576419	0.000516	0.009512	A_23_P6066	GOLD III	CPXM1
1.630261	4.413312	0.000607	0.010551	A_23_P337689	GOLD III	WT1-AS
1.734148	11.64304	0.000635	0.010944	A_23_P148088	GOLD III	FGG
1.442589	4.1529	0.000785	0.012523	A_23_P31755	GOLD III	CRH
1.053707	9.516348	0.00079	0.012546	A_24_P122137	GOLD III	LIF
1.391186	3.489089	0.00079	0.012546	A_23_P116280	GOLD III	WT1
-1.06992	6.228103	0.000958	0.014251	A_24_P242581	GOLD III	SLC5A9
1.968096	5.553758	0.001088	0.015344	A_23_P362694	GOLD III	C4orf7
-1.18434	6.829684	0.001139	0.015821	A_23_P425681	GOLD III	CCK
1.01594	8.511106	0.002893	0.029435	A_23_P81103	GOLD III	SFRP2
1.072239	3.730791	0.003301	0.032217	A_23_P380318	GOLD III	EGR4
1.151253	9.139393	0.005355	0.045099	A_23_P7313	GOLD III	SPP1
1.408752	9.095764	3.43E-16	5.24E-12	A_32_P75581	GOLD IV	BHLHE22
-1.6441	6.625044	2.13E-14	1.63E-10	A_23_P170888	GOLD IV	DPP6
1.311088	11.60193	5.47E-14	2.09E-10	A_23_P209625	GOLD IV	CYP1B1
1.633705	8.904054	1.07E-13	3.26E-10	A_23_P20316	GOLD IV	CA3
1.414833	8.118916	3.55E-13	6.91E-10	A_23_P324754	GOLD IV	KIAA1199
-1.9438	5.023062	4.49E-13	7.08E-10	A_24_P738168	GOLD IV	FREM3
1.470031	4.932203	5.73E-13	7.95E-10	A_23_P256948	GOLD IV	MSC
1.606879	10.48297	8.83E-13	1.12E-09	A_23_P137665	GOLD IV	CHI3L1
1.585092	5.837339	9.84E-13	1.16E-09	A_32_P70315	GOLD IV	TIMP4
1.92926	11.08337	9.80E-12	7.12E-09	A_23_P123853	GOLD IV	CCL19
1.822596	8.087427	1.19E-11	8.26E-09	A_23_P214144	GOLD IV	COL10A1
1.139368	11.89914	3.02E-11	1.59E-08	A_23_P62115	GOLD IV	TIMP1
1.282719	3.858757	3.68E-11	1.87E-08	A_23_P147255	GOLD IV	PCBP3
1.127254	10.48438	4.17E-11	1.99E-08	A_24_P286114	GOLD IV	SLC1A3

logFC	AveExpr	P.Value	adj.P.Val	probe	group	GENE_SYMBOL
-1.41526	3.687686	4.53E-11	2.10E-08	A_24_P174294	GOLD IV	LRRTM4
1.59356	10.28468	5.57E-11	2.30E-08	A_23_P145096	GOLD IV	PLA2G7
1.440505	6.933893	6.51E-11	2.44E-08	A_23_P131024	GOLD IV	ZBTB32
1.783988	5.438078	6.60E-11	2.44E-08	A_24_P252945	GOLD IV	CXCR5
2.009402	5.736843	1.50E-10	4.08E-08	A_23_P13548	GOLD IV	CHRDL2
-1.06663	5.421026	1.61E-10	4.24E-08	A_23_P161135	GOLD IV	LEPR
-1.49337	9.331937	1.66E-10	4.29E-08	A_23_P2674	GOLD IV	KRT4
-1.31162	5.703843	2.22E-10	5.47E-08	A_32_P227605	GOLD IV	RGS9BP
-1.48027	4.263608	2.50E-10	5.96E-08	A_23_P217277	GOLD IV	SLITRK2
-1.13047	4.731736	2.73E-10	6.21E-08	A_23_P354855	GOLD IV	RS1
1.567832	11.71372	3.24E-10	7.06E-08	A_23_P40174	GOLD IV	MMP9
1.720591	6.506955	3.98E-10	8.32E-08	A_23_P31725	GOLD IV	BLK
-1.63284	5.7959	4.50E-10	8.92E-08	A_24_P403150	GOLD IV	FLJ34503
1.756526	8.330826	4.56E-10	8.92E-08	A_23_P113572	GOLD IV	CD19
1.139953	4.615894	5.30E-10	9.91E-08	A_24_P945096	GOLD IV	CACNA1I
1.227128	7.161715	5.78E-10	1.04E-07	A_23_P413760	GOLD IV	P2RX5
1.249806	11.30298	7.52E-10	1.29E-07	A_23_P162918	GOLD IV	SERPINA3
1.079851	11.09119	8.25E-10	1.40E-07	A_23_P343398	GOLD IV	CCR7
1.267499	5.327972	8.77E-10	1.47E-07	A_23_P310274	GOLD IV	PRSS2
1.158874	14.44619	9.14E-10	1.48E-07	A_23_P127584	GOLD IV	NNMT
2.094148	5.403305	1.08E-09	1.74E-07	A_23_P46039	GOLD IV	FCRLA
1.786859	9.514173	1.10E-09	1.75E-07	A_24_P264943	GOLD IV	COMP
-1.14124	6.728884	1.20E-09	1.86E-07	A_23_P17826	GOLD IV	SLC5A1
1.428245	8.377014	1.50E-09	2.17E-07	A_23_P118158	GOLD IV	HS3ST2
1.318612	3.490239	1.92E-09	2.62E-07	A_23_P20311	GOLD IV	DPYS
-1.41125	4.256945	2.04E-09	2.68E-07	A_23_P41789	GOLD IV	SLC27A6
1.001064	7.208741	2.11E-09	2.72E-07	A_23_P16834	GOLD IV	FNDC4
1.031078	10.33384	2.61E-09	3.18E-07	A_23_P23048	GOLD IV	S100A9
1.696565	7.958157	2.83E-09	3.38E-07	A_23_P2645	GOLD IV	SDS
1.346025	6.938401	3.34E-09	3.65E-07	A_23_P39067	GOLD IV	SPIB
1.046565	12.29516	3.99E-09	4.25E-07	A_23_P108751	GOLD IV	FHL2
1.367405	7.537411	4.43E-09	4.60E-07	A_23_P253321	GOLD IV	PNOC
1.552449	11.06426	4.91E-09	5.00E-07	A_23_P312920	GOLD IV	POU2AF1
-1.07725	5.814624	5.76E-09	5.63E-07	A_23_P382240	GOLD IV	TMEM26
2.04681	9.286523	6.72E-09	6.37E-07	A_23_P121064	GOLD IV	PTX3
-1.86335	9.244094	8.42E-09	7.47E-07	A_23_P145841	GOLD IV	SOSTDC1
1.83263	6.654159	8.44E-09	7.47E-07	A_23_P165598	GOLD IV	DAPL1
-1.769	5.73862	9.05E-09	7.73E-07	A_32_P8221	GOLD IV	GRM8
-1.23993	13.54074	9.11E-09	7.73E-07	A_23_P72697	GOLD IV	GPIHBP1

logFC	AveExpr	P.Value	adj.P.Val	probe	group	GENE_SYMBOL
1.593133	7.835862	1.01E-08	8.30E-07	A_23_P201211	GOLD IV	FCRL5
-1.18597	10.50269	1.07E-08	8.74E-07	A_23_P113793	GOLD IV	ZBED2
1.590226	6.845509	1.65E-08	1.21E-06	A_23_P85209	GOLD IV	IL13RA2
-1.1833	3.85363	2.01E-08	1.44E-06	A_24_P37253	GOLD IV	LYPD6
1.614068	5.40992	2.38E-08	1.63E-06	A_24_P63019	GOLD IV	IL1R2
1.256698	6.700926	2.65E-08	1.77E-06	A_23_P127288	GOLD IV	IL2RA
1.938453	3.76122	2.90E-08	1.86E-06	A_23_P432947	GOLD IV	GREM1
1.106051	9.76238	3.10E-08	1.95E-06	A_23_P31453	GOLD IV	STEAP1
1.786101	4.160952	3.28E-08	2.05E-06	A_23_P90888	GOLD IV	CHRNA1
1.680189	7.567848	3.30E-08	2.05E-06	A_23_P77493	GOLD IV	TUBB3
-1.07488	7.074765	4.35E-08	2.62E-06	A_24_P13024	GOLD IV	SLC16A12
1.11892	5.508057	4.90E-08	2.85E-06	A_23_P140760	GOLD IV	GPR97
-1.1082	5.287635	5.27E-08	2.99E-06	A_23_P433050	GOLD IV	RXFP1
-1.24301	7.306775	5.43E-08	3.06E-06	A_24_P366607	GOLD IV	SERTM1
-1.0588	3.865826	6.00E-08	3.32E-06	A_23_P368779	GOLD IV	ZNF114
-1.3683	8.64565	6.93E-08	3.66E-06	A_23_P65307	GOLD IV	SLITRK6
-1.03191	9.97349	8.54E-08	4.35E-06	A_23_P31376	GOLD IV	LRRN3
-1.05128	3.033148	9.44E-08	4.65E-06	A_24_P285055	GOLD IV	PCDH11Y
2.108961	7.724092	1.07E-07	5.13E-06	A_23_P1691	GOLD IV	MMP1
-1.44838	4.794553	1.54E-07	6.74E-06	A_24_P349117	GOLD IV	GPR158
1.030878	6.20735	1.68E-07	7.26E-06	A_23_P25155	GOLD IV	GPR84
1.065017	8.823965	1.69E-07	7.29E-06	A_23_P58251	GOLD IV	CPZ
2.008632	4.1529	1.92E-07	7.99E-06	A_23_P31755	GOLD IV	CRH
1.423956	5.344365	2.47E-07	9.67E-06	A_23_P372946	GOLD IV	TM4SF19
-1.18066	10.94223	2.50E-07	9.75E-06	A_24_P13041	GOLD IV	RTKN2
1.072508	8.126823	3.00E-07	1.12E-05	A_23_P114983	GOLD IV	TRIM63
1.520341	5.106319	3.01E-07	1.12E-05	A_23_P204947	GOLD IV	GJB2
1.447237	9.405814	3.79E-07	1.33E-05	A_23_P74001	GOLD IV	S100A12
1.483504	3.907201	3.88E-07	1.35E-05	A_23_P152262	GOLD IV	DPEP1
-1.04103	6.899158	3.95E-07	1.37E-05	A_32_P213459	GOLD IV	DMRT2
1.886012	9.139393	3.99E-07	1.37E-05	A_23_P7313	GOLD IV	SPP1
-1.04216	9.585841	5.51E-07	1.77E-05	A_24_P71904	GOLD IV	HPGD
1.402998	9.516348	6.08E-07	1.91E-05	A_24_P122137	GOLD IV	LIF
1.033895	5.536251	6.33E-07	1.97E-05	A_24_P288890	GOLD IV	FAM101A
-1.06204	4.57606	8.40E-07	2.42E-05	A_23_P347777	GOLD IV	SYCP2L
1.917719	8.420819	9.31E-07	2.63E-05	A_24_P335092	GOLD IV	SAA1
1.013934	7.346549	9.86E-07	2.77E-05	A_23_P115161	GOLD IV	DARC
-1.02547	9.578368	1.03E-06	2.86E-05	A_23_P129144	GOLD IV	MYZAP
-1.01692	7.264003	1.05E-06	2.90E-05	A_23_P68219	GOLD IV	TTN

logFC	AveExpr	P.Value	adj.P.Val	probe	group	GENE_SYMBOL
1.142149	10.68325	1.13E-06	3.08E-05	A_23_P144916	GOLD IV	GFPT2
1.27554	10.6328	1.26E-06	3.34E-05	A_23_P84596	GOLD IV	MZB1
-1.13921	4.679222	1.59E-06	3.96E-05	A_23_P18362	GOLD IV	SLITRK3
1.186721	11.84822	1.94E-06	4.60E-05	A_24_P142118	GOLD IV	THBS1
-1.37549	6.228103	2.04E-06	4.79E-05	A_24_P242581	GOLD IV	SLC5A9
1.320644	3.126064	2.27E-06	5.16E-05	A_24_P917819	GOLD IV	ANKRD30BP2
2.122791	11.64304	2.80E-06	6.01E-05	A_23_P148088	GOLD IV	FGG
2.277401	5.449969	2.83E-06	6.05E-05	A_23_P121695	GOLD IV	CXCL13
1.202338	3.139875	2.89E-06	6.13E-05	A_32_P345659	GOLD IV	XIRP1
1.455181	10.40163	3.46E-06	7.00E-05	A_23_P166408	GOLD IV	OSM
-1.24275	8.453668	3.59E-06	7.23E-05	A_23_P81280	GOLD IV	BTNL9
1.435399	7.373083	4.19E-06	8.14E-05	A_23_P148737	GOLD IV	MYBPH
1.310734	4.505471	4.20E-06	8.15E-05	A_23_P146274	GOLD IV	STMN2
1.231771	4.759306	4.44E-06	8.54E-05	A_23_P421306	GOLD IV	SYT12
1.018978	9.128814	4.80E-06	9.03E-05	A_24_P37409	GOLD IV	DUSP2
1.201676	6.400447	5.04E-06	9.36E-05	A_23_P57417	GOLD IV	MMP11
1.022567	3.382741	5.07E-06	9.39E-05	A_23_P84705	GOLD IV	TNFRSF13B
1.026541	7.077879	7.27E-06	0.000123	A_23_P160159	GOLD IV	SLC2A5
1.925543	9.682829	7.69E-06	0.000129	A_23_P126278	GOLD IV	CHIT1
1.010616	8.099627	8.14E-06	0.000136	A_24_P335620	GOLD IV	SLC7A5
-1.19449	11.80404	8.89E-06	0.000147	A_24_P53778	GOLD IV	ITLN2
-1.00759	5.497394	9.54E-06	0.000155	A_23_P401774	GOLD IV	ELMOD1
1.189199	10.8577	1.18E-05	0.000183	A_23_P58266	GOLD IV	S100P
1.040879	5.348151	1.18E-05	0.000183	A_24_P98047	GOLD IV	SLC16A10
1.056437	4.271359	1.59E-05	0.000229	A_23_P208747	GOLD IV	PGLYRP1
1.532943	3.663398	1.80E-05	0.000254	A_23_P22134	GOLD IV	BNC1
1.071739	14.9849	1.92E-05	0.000267	A_23_P120812	GOLD IV	IGLL1
-1.02871	8.494428	1.99E-05	0.000275	A_24_P244706	GOLD IV	SSTR1
1.229686	5.345542	2.11E-05	0.000287	A_32_P3476	GOLD IV	RPRML
1.631576	5.573696	2.64E-05	0.000337	A_23_P380240	GOLD IV	CEACAM8
1.337572	7.465669	3.02E-05	0.000375	A_23_P78248	GOLD IV	KRT23
-1.01906	6.31235	3.08E-05	0.00038	A_32_P182299	GOLD IV	C1orf168
1.292874	8.643536	3.08E-05	0.000381	A_24_P97342	GOLD IV	PROK2
-1.10118	3.456277	3.40E-05	0.00041	A_23_P144656	GOLD IV	CDH10
1.104211	6.782847	3.77E-05	0.000445	A_23_P66635	GOLD IV	CCL11
1.005115	6.035169	4.15E-05	0.000481	A_23_P42897	GOLD IV	MGAM
1.050453	5.678478	4.49E-05	0.000513	A_24_P125469	GOLD IV	LIPG
1.778762	6.972516	4.68E-05	0.000529	A_23_P96158	GOLD IV	KRT17
1.028945	6.720952	4.75E-05	0.000534	A_23_P67932	GOLD IV	CXCR1

logFC	AveExpr	P.Value	adj.P.Val	probe	group	GENE_SYMBOL
1.02871	5.600271	5.17E-05	0.00057	A_23_P69537	GOLD IV	NMU
1.079319	14.08995	5.32E-05	0.000584	A_23_P206760	GOLD IV	HP
1.088267	5.694138	7.01E-05	0.000729	A_23_P79769	GOLD IV	BIRC7
1.162448	11.18291	7.10E-05	0.000737	A_23_P86470	GOLD IV	CH25H
2.11837	5.553758	7.46E-05	0.000767	A_23_P362694	GOLD IV	C4orf7
1.732555	5.342859	0.000102	0.000988	A_23_P375372	GOLD IV	FGA
-1.90622	4.434363	0.000106	0.00102	A_32_P780817	GOLD IV	CT45A1
1.307362	3.042065	0.000114	0.001071	A_23_P366936	GOLD IV	KRT6C
1.05652	4.056378	0.000126	0.001156	A_23_P138262	GOLD IV	PADI4
1.978736	10.95013	0.00013	0.001184	A_23_P360754	GOLD IV	ADAMTS4
-1.20387	4.010685	0.000149	0.001316	A_23_P161659	GOLD IV	SYT13
1.128648	5.786577	0.000182	0.001529	A_24_P95723	GOLD IV	KIAA0125
1.047276	6.298834	0.000227	0.001812	A_23_P386478	GOLD IV	TNIP3
1.342241	4.130889	0.000231	0.001839	A_23_P78037	GOLD IV	CCL7
1.000967	2.658809	0.000261	0.002029	A_24_P245379	GOLD IV	SERPINB2
1.407327	6.50973	0.00031	0.002331	A_23_P128744	GOLD IV	BDKRB1
1.246288	4.266623	0.000312	0.002337	A_23_P38537	GOLD IV	KRT16
1.037073	6.576419	0.000331	0.002452	A_23_P6066	GOLD IV	CPXM1
1.132649	5.378316	0.000377	0.002718	A_23_P146146	GOLD IV	ATP6V0D2
1.483196	7.152695	0.000383	0.002747	A_23_P321949	GOLD IV	PLA2G2A
1.275024	9.303138	0.000394	0.002815	A_23_P99515	GOLD IV	C13orf33
-1.42848	5.569232	0.000465	0.003213	A_32_P170547	GOLD IV	CT45A5
1.000409	4.972083	0.000497	0.003364	A_23_P134914	GOLD IV	LY6H
1.097623	3.019588	0.000553	0.00365	A_24_P200219	GOLD IV	UPK1B
1.020823	5.611813	0.000779	0.004761	A_23_P161624	GOLD IV	FOSL1
1.352479	4.00174	0.001192	0.00657	A_23_P13094	GOLD IV	MMP10
-1.10692	5.312968	0.001321	0.007087	A_23_P7727	GOLD IV	HAPLN1
1.424236	7.855309	0.001339	0.007161	A_32_P87013	GOLD IV	IL8
1.090623	3.727838	0.002583	0.012079	A_24_P174793	GOLD IV	PCSK1
1.255497	4.413312	0.002708	0.012507	A_23_P337689	GOLD IV	WT1-AS
1.581507	5.349175	0.002727	0.012583	A_23_P27400	GOLD IV	HAS1
1.087614	3.489089	0.00289	0.013155	A_23_P116280	GOLD IV	WT1
1.325086	11.18496	0.00449	0.01848	A_23_P71037	GOLD IV	IL6
1.391156	8.4265	0.004824	0.019568	A_23_P97112	GOLD IV	SELE
1.526658	10.78351	0.007153	0.026729	A_23_P95790	GOLD IV	ITLN1
1.323148	6.16776	0.008728	0.031423	A_23_P218047	GOLD IV	KRT5
1.005627	6.267205	0.011343	0.038502	A_23_P306867	GOLD IV	NR4A3

Table S1 Differentially expressed genes (DEG) in gold I to IV patients vs healthy controls

Table S2. Sources for omics data

Omics Type	Tissue	Reference	N subjects*
Transcriptomics	Lung	Tan et al., 2016	328
	Lung	Morrow et al., 2017	150
Proteomics	Lung	Yu-Hang Zhang et al., 2023	98
	Lung	Ohlmeier et al., 2016	10
	Lung	Barrero et al., 2013	25
	Lung	Lee et al., 2009	14
	Lung	Ohlmeier et al., 2008	8
Metabolomics	Bronchoalveolar Lavage Fluid	Halper-Stromberg et al., 2019	115
Genomics	Blood	Shrine et al., 2023 (Meta analysis)	588,452

*Include both COPD and healthy control subjects.

Table S2. Sources for omics data

Table S3. Summary of the variant-to-gene mapping evidence for the 135 suspected genes associated with COPD

Evidence Type	N gene (Total = 135)	Percentage (n/135)
the nearest gene to the sentinel SNP	131	97.0
polygenic priority score	122	90.4
co-localization of the GWAS signal and expression quantitative trait loci (eQTL)	110	81.5
proximity to a gene for a Mendelian disease with a respiratory phenotype (±500 kb)	22	16.3
annotation-informed credible sets: functional GWAS analysis	20	14.8
rare variant association in whole-exome sequencing in the UK Biobank	11	8.1
nearby mouse knockout orthologs with a respiratory phenotype	5	3.7
protein quantitative trait loci (pQTL) signals in relevant tissues	5	3.7

*For more detailed information on the specific SNPs and genes, please refer to the Supplementary Table 13 in the original paper from Shrine et al [49].

Table S3: Summary of the variant-to-gene mapping evidence for the 135 suspected genes associated with COPD

Table S4. Proteomics studies on lung tissues from COPD patients

Year	Authors	Tissue	Groups	Differentially expressed proteins (shown as the corresponding gene symbols)
2023	Zhang, Yu-Hang, et al	Lung	Healthy smokers vs COPD patients	EHD3, FOLR1, GPRC5A, AQP4, FTL, PGD, LDHA, ESAM, RDX, FTH1, CALCRL, AGER, MZB1, CAP1, PPIB, PPIL3, AQP1, COLGALT1, SPARCL1, PCYOX1, CA3, CAV1, HSP90B1, LGMN, SUSD2, COL14A1, HPGD, PLCB3, EHD2, RALA, LTC4S, PPP1CC, TGM2, ENO1, LCP1, IFIT3, P4HB, CCAR2, VAMP3, PLLP, LAMA4, COL12A1, ANO6, GABARAP, FARP1, ALCAM, BPIFA1, HPCAL1, S100A10, ARRB1, CDH1, CAVIN2, PSMD5, PCDH1, MAPK3
2013	Barrero, Carlos A., et al	Lung	Healthy smokers vs COPD GOLD IV patients	H3F3B/H3F3A, H2BFS, H4C1, ICAM1, MES, MYH9, MYH10 , VIM , FIBA , FIBG , EHD2 , TLN1 , PERM1 , DHE3, ATP1A1 , TGM2 , COL6A3
2016	Ohlmeier, Steffen, et al	Lung	Healthy smokers vs GOLD I-II patients	CTSD, TGM2, TPP1, FBP1, COL6A1, EEF1G, MYL6, ASAH1, FTL, PRDX3, SERPINB1, TPM1, UQCRC1, AMBP, APOA1, HBB, ITIH4, TF
			Healthy smokers vs COPD III-IV patients	TGM2, S100A4, ACTN1, ACTA2, CCT5, COL6A1, EEF1G, HSPB1, HSP90AB1, MFAP4, MYL6, NAPA, TPM1, TPM2, USP5, ANXA7, PTRF, TPM4, UQCRC1, AMBP, APOA1, TF, CLU
2009	Lee, Eun Joo, et al	Lung	Healthy smokers vs COPD patients	MMP13, TFAP4, PSME3IP1, HDGF, NAPA, CETN3, CAV1, GLRX3, TPT1, CALML3
2008	Ohlmeier, Steffen, et al	Lung	Healthy controls (never smokers and ex-smokers) vs COPD IV patients	SFTPA2/SFTPA1

Table S4: Proteomics studies on lung tissues from COPD patients

Table S5. Metabolites input for MAGI

Compound	inchi key (MAGI input)	searched by MAGI*
Mycalamide B	AAABMNXUOFPYQK-GRMLSQNXSA-N	No
S-(Phenylacetothiohydroximoyl)-L-cysteine	DPHQZNQYOOCWSR-CVZWZAMJSA-N	No
Homocysteine	FFFHZYDWPBMWHY-VKHMYHEASA-N	Yes
Lophocerine	GEHUGSUAESFIIV-UHFFFAOYSA-N	Yes
L-Threonylcarbamoyladenylate	GHLUPQUHEIJRCU-DWVDDHQFSA-N	Yes
p-cresol	IWDCLRJOBJJRNH-UHFFFAOYSA-N	Yes
N-palmitoyl glycine	KVTFEOAKFFQCCX-UHFFFAOYSA-N	Yes
Glycocholic acid	RFDAIACWWDREDC-FRVQLJSFSA-N	Yes
SM (d18:1/24:1)	WKZHECFHXLTOLJ-QYKFWSDSSA-N	Yes
Decaprenyl phosphate	XBEJBEIXLWRYBT-DJNGBRKISA-N	Yes
Ceramide (d18:1/16:0)	YDNKGFDKKRUKPY-TURZORIXSA-N	Yes
PS (37:3)	GTQLLUSBBJKGBX-WWYFVLCKSA-N	No
PE (38:3)	MSYFXMJDQLPRGO-JTBMWNAQSA-N	No
PC(40:6)	TXHZYNSTTCIWMJ-SQKXFFESSA-N	No
PC (40:6) (isomer)	KZSXWBSDQDXESU-PPEVQBTRSA-N	Yes
PC (32:1)	QIBZFHLFHCIUOT-ZPHPHTNESA-N	No
MGDG (36:5)	DRLQFBRXASRGDP-XJYZHZQCSA-N	Yes
PE (35:1)	NDZIIJNXYISRGX-YWTUKGCKSA-N	Yes
PC (36:4)	NKQPOVROGSWLTO-NVPMBMBWSA-N	Yes
PE (36:3)	LMWFNZUKABEGHS-CISNCOODSA-N	Yes
PC (34:2)	JLPULHDHAOZNQI-UHFFFAOYSA-O	No
SQMG (16:1)	OGARSMGVMPSJRA-FEASXFNBSA-N	Yes
PE (34:2)	HBZNVZIRJWODIB-NHCUFCNUSA-N	Yes

CL (70:0)	HIPHRGUIEMLVAI-UHFFFAOYSA-N	Yes
CL (72:7)	ZGKKGDOIMJSRJH-YHMFKNNJSA-N	No

*Compounds not in MAGI database were not searched

Table S5 Metabolites input for MAGI

Table S6. Signature genes identified on more than one omics levels

	Genomics	Transcriptomics		Proteomics	
Gene	p_min	fold change	adjusted p value	fold change	p value
AGER	1.5E-227	0.60	2.18E-04	0.55	4.16E-05
CA3	NA	3.10	3.26E-10	1.68	1.43E-04
DMRT2	4.48E-15	0.49	1.37E-05	NA	NA
HPGD	NA	0.49	1.77E-05	0.46	1.59E-04
MZB1	NA	2.42	3.34E-05	2.99	5.04E-05
THBS1	2.61E-11	2.28	4.60E-05	NA	NA
TPM1	6.88E-10	1.00	9.77E-01	3.91	1.65E-03
TTN	2.08E-11	0.49	2.90E-05	NA	NA

Note: In genomics data, multiple variants can be mapped to the same gene. The p value of the most significant variant implicating the gene is reported in the table based on its association with the lung function measured by FEV1/FVC. In the proteomics data, nominal p-values are provided due to the lack of raw data.

Table S6: number of COPD studies involved with drugs targeting the tier 1 druggable genes

Table S7. Significantly enriched pathways for the 92 COPD signature genes

ID	Description	p.adjust	geneID
GO:0030198	extracellular matrix organization	1.9E-09	ADAMTS4/COL10A1/COL12A1/COL14A1/COL4A2/COLGALT1/IL6/LAMA2/LRP1/MMP1/MMP13/MMP9/RXFP1/SMAD3/TGFB2/WT1
GO:0043062	extracellular structure organization	1.9E-09	ADAMTS4/COL10A1/COL12A1/COL14A1/COL4A2/COLGALT1/IL6/LAMA2/LRP1/MMP1/MMP13/MMP9/RXFP1/SMAD3/TGFB2/WT1
GO:0045229	external encapsulating structure organization	1.9E-09	ADAMTS4/COL10A1/COL12A1/COL14A1/COL4A2/COLGALT1/IL6/LAMA2/LRP1/MMP1/MMP13/MMP9/RXFP1/SMAD3/TGFB2/WT1
GO:0050900	leukocyte migration	3.7E-08	AGER/ANO6/BDKRB1/CCL11/CXCR1/CXCR5/IL6/ITGA1/MAPK3/MMP9/MYH9/PPIB/S100A12/SAA1/TGFB2/THBS1
GO:0035270	endocrine system development	4.4E-07	APOA1/BRAF/CDH1/CRH/IL6/MAPK3/NR3C1/SMAD3/TBX1/WT1
GO:0043410	positive regulation of MAPK cascade	5.4E-07	ADRB2/AGER/ARRB1/BRAF/CCL11/CSK/FGA/FGG/IGF1R/IL6/ITGA1/MAPK3/S100A12/TBX1/TGFB2/THBS1
GO:0030595	leukocyte chemotaxis	5.4E-07	ANO6/CCL11/CXCR1/CXCR5/IL6/ITGA1/MAPK3/PPIB/S100A12/SAA1/TGFB2/THBS1
GO:0097529	myeloid leukocyte migration	5.4E-07	AGER/ANO6/CCL11/CXCR1/IL6/ITGA1/MAPK3/PPIB/S100A12/SAA1/TGFB2/THBS1
GO:0045785	positive regulation of cell adhesion	2.4E-06	AGER/AP3B1/APOA1/BRAF/CSK/FGA/FGG/IL6/ITGAV/LAMA2/P4HB/SAA1/SMAD3/TGFB2/TPM1
GO:0006936	muscle contraction	2.7E-06	ADRB2/ATP1A1/CALCRL/CHRM3/CHRNA1/CTTN/MYBPH/NMU/PROK2/TPM1/TPM2/TPM4/TTN
GO:0071621	granulocyte chemotaxis	2.7E-06	CCL11/CXCR1/ITGA1/MAPK3/PPIB/S100A12/SAA1/TGFB2/THBS1
GO:0001503	ossification	3.1E-06	ADRB2/ANO6/COL6A1/FHL2/IL6/MAPK3/MMP13/MMP9/RUNX2/SMAD3/SPP1/TGFB2/TPM4/TWIST2
GO:0042060	wound healing	4.2E-06	ANO6/AP3B1/FGA/FGG/HBB/IL6/MYH9/SAA1/SMAD3/TGFB2/THBS1/TIMP1/TLN1/TPM1
GO:0003012	muscle system process	5.6E-06	ADRB2/ATP1A1/CALCRL/CHRM3/CHRNA1/CTTN/MYBPH/NMU/PROK2/SMAD3/TPM1/TPM2/TPM4/TTN
GO:0060326	cell chemotaxis	6.3E-06	ANO6/CCL11/CXCR1/CXCR5/IL6/ITGA1/MAPK3/PPIB/S100A12/SAA1/TGFB2/THBS1
GO:0097530	granulocyte migration	1.0E-05	CCL11/CXCR1/ITGA1/MAPK3/PPIB/S100A12/SAA1/TGFB2/THBS1
GO:0007596	blood coagulation	1.8E-05	ANO6/AP3B1/FGA/FGG/HBB/IL6/MYH9/SAA1/THBS1/TLN1

ID	Description	p.adjust	geneID
GO:0050817	coagulation	1.9E-05	ANO6/AP3B1/FGA/FGG/HBB/IL6/MYH9/SAA1/THBS1/TLN1
GO:0006937	regulation of muscle contraction	1.9E-05	ADRB2/ATP1A1/CALCRL/CHRM3/CTTN/MYBPH/NMU/PROK2/TPM1
GO:0007599	hemostasis	1.9E-05	ANO6/AP3B1/FGA/FGG/HBB/IL6/MYH9/SAA1/THBS1/TLN1
GO:0030325	adrenal gland development	1.9E-05	APOA1/CRH/NR3C1/SMAD3/WT1
GO:0048732	gland development	1.9E-05	APOA1/BRAF/CCL11/CDH1/CRH/IL6/MAPK3/NR3C1/RXFP1/SMAD3/TBX1/TGFB2/WT1
GO:0031589	cell-substrate adhesion	2.0E-05	ACTN1/APOA1/BRAF/CTTN/FGA/FGG/ITGA1/ITGAV/P4HB/SMAD3/THBS1/TLN1
GO:0007015	actin filament organization	2.4E-05	ACTN1/APOA1/ARRB1/BRAF/CCL11/CTTN/HSP90B1/SMAD3/TF/TPM1/TPM2/TPM4/TTN
GO:1904645	response to amyloid-beta	2.4E-05	ADRB2/AGER/IGF1R/LRP1/MMP13/MMP9
GO:1904019	epithelial cell apoptotic process	2.9E-05	ANO6/BCL2L1/BRAF/FGA/FGG/IGF1R/IL6/THBS1
GO:0090257	regulation of muscle system process	3.7E-05	ADRB2/ATP1A1/CALCRL/CHRM3/CTTN/MYBPH/NMU/PROK2/SMAD3/TPM1
GO:0022617	extracellular matrix disassembly	4.5E-05	ADAMTS4/IL6/LRP1/MMP1/MMP13/MMP9
GO:0071674	mononuclear cell migration	5.3E-05	AGER/ANO6/CCL11/CXCR1/IL6/MAPK3/S100A12/SAA1/THBS1
GO:0006940	regulation of smooth muscle contraction	5.5E-05	ADRB2/CALCRL/CHRM3/CTTN/NMU/PROK2
GO:0030593	neutrophil chemotaxis	6.0E-05	CCL11/CXCR1/ITGA1/PPIB/S100A12/SAA1/TGFB2
GO:1904035	regulation of epithelial cell apoptotic process	7.5E-05	ANO6/BRAF/FGA/FGG/IGF1R/IL6/THBS1
GO:0070527	platelet aggregation	8.2E-05	FGA/FGG/HBB/IL6/MYH9/TLN1
GO:0071634	regulation of transforming growth factor beta production	8.2E-05	ITGAV/LTBP1/SMAD3/TGFB2/THBS1
GO:0007229	integrin-mediated signaling pathway	8.2E-05	APOA1/ITGA1/ITGAV/LAMA2/MYH9/TIMP1/TLN1
GO:0071604	transforming growth factor beta production	1.1E-04	ITGAV/LTBP1/SMAD3/TGFB2/THBS1
GO:1903034	regulation of response to wounding	1.1E-04	ANO6/BRAF/FGA/FGG/IGF1R/SMAD3/SPP1/THBS1
GO:1902905	positive regulation of supramolecular fiber organization	1.3E-04	APOA1/BRAF/CCL11/CLU/COLGALT1/CTTN/SMAD3/TPM1
GO:0007160	cell-matrix adhesion	1.3E-04	ACTN1/CTTN/FGA/FGG/ITGA1/ITGAV/SMAD3/THBS1/TLN1

ID	Description	p.adjust	geneID
GO:1900026	positive regulation of substrate adhesion-dependent cell spreading	1.3E-04	APOA1/BRAF/FGA/FGG/P4HB
GO:0050878	regulation of body fluid levels	1.5E-04	ANO6/AP3B1/CHRM3/FGA/FGG/HBB/IL6/MYH9/SAA1/THBS1/TLN1
GO:0045056	transcytosis	1.6E-04	AGER/GPIHBP1/IGF1R/LRP1
GO:0007492	endoderm development	1.6E-04	COL12A1/COL4A2/COL6A1/ITGAV/MMP9/SMAD3
GO:1990266	neutrophil migration	1.7E-04	CCL11/CXCR1/ITGA1/PPIB/S100A12/SAA1/TGFB2
GO:0030168	platelet activation	1.9E-04	FGA/FGG/HBB/IL6/MYH9/SAA1/TLN1
GO:0035987	endodermal cell differentiation	1.9E-04	COL12A1/COL4A2/COL6A1/ITGAV/MMP9
GO:0045933	positive regulation of muscle contraction	1.9E-04	ATP1A1/CHRM3/CTTN/NMU/PROK2
GO:0008217	regulation of blood pressure	1.9E-04	ADRB2/ATP1A1/BDKRB1/HBB/PLCB3/SLC2A5/SMAD3/TPM1
GO:0006898	receptor-mediated endocytosis	2.0E-04	ADRB2/ARRB1/CALCRL/CLU/CTTN/CXCR1/ITGAV/LRP1/TF
GO:0070371	ERK1 and ERK2 cascade	2.5E-04	AGER/ARRB1/BRAF/CCL11/CSK/FGA/FGG/ITGAV/MAPK3/TF
GO:0008211	glucocorticoid metabolic process	2.8E-04	APOA1/ATP1A1/CRH/NR3C1
GO:0034114	regulation of heterotypic cell-cell adhesion	2.8E-04	AGER/APOA1/FGA/FGG
GO:0031032	actomyosin structure organization	2.8E-04	APOA1/ARRB1/BRAF/MYH10/MYH9/SMAD3/TPM1/TTN
GO:0034109	homotypic cell-cell adhesion	2.9E-04	FGA/FGG/HBB/IL6/MYH9/TLN1
GO:1903053	regulation of extracellular matrix organization	3.5E-04	COLGALT1/IL6/LAMA2/LRP1/SMAD3
GO:2001237	negative regulation of extrinsic apoptotic signaling pathway	3.5E-04	BCL2L1/CTTN/FGA/FGG/ITGAV/THBS1
GO:0001706	endoderm formation	3.7E-04	COL12A1/COL4A2/COL6A1/ITGAV/MMP9
GO:0030878	thyroid gland development	4.0E-04	BRAF/MAPK3/SMAD3/TBX1
GO:2000351	regulation of endothelial cell apoptotic process	4.2E-04	ANO6/BRAF/FGA/FGG/THBS1
GO:0034113	heterotypic cell-cell adhesion	4.4E-04	AGER/APOA1/FGA/FGG/ITGAV

ID	Description	p.adjust	geneID
GO:1900024	regulation of substrate adhesion-dependent cell spreading	4.4E-04	APOA1/BRAF/FGA/FGG/P4HB
GO:0010810	regulation of cell-substrate adhesion	4.5E-04	APOA1/BRAF/FGA/FGG/P4HB/SMAD3/THBS1/TLN1
GO:0051017	actin filament bundle assembly	4.6E-04	ACTN1/APOA1/ARRB1/BRAF/HSP90B1/SMAD3/TPM1
GO:0097191	extrinsic apoptotic signaling pathway	4.6E-04	BCL2L1/CTTN/FGA/FGG/ITGAV/SMAD3/TGFB2/THBS1
GO:0045453	bone resorption	4.9E-04	ADRB2/CSK/IL6/SPP1/TF
GO:0061572	actin filament bundle organization	5.2E-04	ACTN1/APOA1/ARRB1/BRAF/HSP90B1/SMAD3/TPM1
GO:0045987	positive regulation of smooth muscle contraction	5.2E-04	CHRM3/CTTN/NMU/PROK2
GO:0034446	substrate adhesion-dependent cell spreading	5.6E-04	APOA1/BRAF/FGA/FGG/ITGAV/P4HB
GO:2001234	negative regulation of apoptotic signaling pathway	5.7E-04	BCL2L1/CLU/CTTN/FGA/FGG/ITGAV/MMP9/THBS1
GO:0072577	endothelial cell apoptotic process	5.7E-04	ANO6/BRAF/FGA/FGG/THBS1
GO:0007178	transmembrane receptor protein serine/threonine kinase signaling pathway	5.9E-04	FBN1/LRP1/LTBP1/MAPK3/RUNX2/SMAD3/TF/TGFB2/THBS1/VIM
GO:0006939	smooth muscle contraction	5.9E-04	ADRB2/CALCRL/CHRM3/CTTN/NMU/PROK2
GO:0071492	cellular response to UV-A	5.9E-04	MMP1/MMP9/TIMP1
GO:0022604	regulation of cell morphogenesis	6.1E-04	APOA1/BRAF/CCL11/FGA/FGG/MYH10/MYH9/P4HB/TPM1
GO:0001649	osteoblast differentiation	7.8E-04	COL6A1/FHL2/IL6/RUNX2/SMAD3/SPP1/TPM4/TWIST2
GO:0150076	neuroinflammatory response	8.5E-04	AGER/CLU/IL6/LRP1/MMP9
GO:0030278	regulation of ossification	8.5E-04	ADRB2/ANO6/MAPK3/RUNX2/SMAD3/TGFB2
GO:1905952	regulation of lipid localization	9.5E-04	APOA1/CRH/IL6/ITGAV/LRP1/SPP1/THBS1

ID	Description	p.adjust	geneID
GO:0010811	positive regulation of cell-substrate adhesion	9.5E-04	APOA1/BRAF/FGA/FGG/P4HB/SMAD3
GO:0001704	formation of primary germ layer	1.0E-03	COL12A1/COL4A2/COL6A1/ITGAV/MMP9/SMAD3
GO:0070141	response to UV-A	1.2E-03	MMP1/MMP9/TIMP1
GO:0007517	muscle organ development	1.3E-03	CHRNA1/COL6A3/LAMA2/SMAD3/TBX1/TGFB2/TPM1/TTN/WT1
GO:0007369	gastrulation	1.3E-03	APOA1/COL12A1/COL4A2/COL6A1/ITGAV/MMP9/SMAD3
GO:0045124	regulation of bone resorption	1.3E-03	CSK/IL6/SPP1/TF
GO:0003018	vascular process in circulatory system	1.3E-03	ADRB2/AGER/CHRM3/FGA/FGG/HBB/ITGA1/LRP1
GO:0010770	positive regulation of cell morphogenesis involved in differentiation	1.3E-03	APOA1/BRAF/FGA/FGG/P4HB
GO:0048708	astrocyte differentiation	1.3E-03	AGER/IL6/LRP1/MAPK3/VIM
GO:0060395	SMAD protein signal transduction	1.3E-03	LRP1/SMAD3/TF/TGFB2/VIM
GO:0071560	cellular response to transforming growth factor beta stimulus	1.3E-03	COL4A2/FBN1/IGF1R/LTBP1/NR3C1/SMAD3/TGFB2/THBS1
GO:0034116	positive regulation of heterotypic cell-cell adhesion	1.3E-03	AGER/FGA/FGG
GO:0014002	astrocyte development	1.3E-03	AGER/IL6/LRP1/VIM
GO:0071559	response to transforming growth factor beta	1.5E-03	COL4A2/FBN1/IGF1R/LTBP1/NR3C1/SMAD3/TGFB2/THBS1
GO:0048041	focal adhesion assembly	1.5E-03	ACTN1/CTTN/SMAD3/THBS1/TLN1
GO:0010038	response to metal ion	1.5E-03	BRAF/CDH1/FGA/FGG/MAPK3/MMP9/TF/THBS1/TTN
GO:1901654	response to ketone	1.5E-03	BCL2L1/GNB1/IGF1R/NR3C1/SPP1/TGFB2/THBS1
GO:0006704	glucocorticoid biosynthetic process	1.5E-03	ATP1A1/CRH/NR3C1
GO:0090030	regulation of steroid hormone biosynthetic process	1.5E-03	ATP1A1/IGF1R/NR3C1
GO:1904646	cellular response to amyloid-beta	1.6E-03	ADRB2/AGER/IGF1R/LRP1

ID	Description	p.adjust	geneID
GO:0071692	protein localization to extracellular region	1.7E-03	FBN1/FGA/FGG/IL6/LTBP1/MYH10/SAA1/TGFB2/TTN
GO:0050921	positive regulation of chemotaxis	1.7E-03	AGER/ANO6/IL6/MAPK3/SMAD3/THBS1
GO:0046849	bone remodeling	1.7E-03	ADRB2/CSK/IL6/SPP1/TF
GO:0035296	regulation of tube diameter	1.8E-03	ADRB2/CHRM3/FGA/FGG/HBB/ITGA1
GO:0097746	blood vessel diameter maintenance	1.8E-03	ADRB2/CHRM3/FGA/FGG/HBB/ITGA1
GO:2001233	regulation of apoptotic signaling pathway	1.8E-03	BCL2L1/CLU/CTTN/FGA/FGG/ITGAV/MMP9/P4HB/THBS1
GO:1902895	positive regulation of miRNA transcription	1.8E-03	NR3C1/SMAD3/TGFB2/WT1
GO:0002687	positive regulation of leukocyte migration	1.8E-03	AGER/ANO6/BDKRB1/IL6/MAPK3/THBS1
GO:0035150	regulation of tube size	1.8E-03	ADRB2/CHRM3/FGA/FGG/HBB/ITGA1
GO:0046850	regulation of bone remodeling	1.9E-03	CSK/IL6/SPP1/TF
GO:1903035	negative regulation of response to wounding	2.0E-03	FGA/FGG/SMAD3/SPP1/THBS1
GO:0007044	cell-substrate junction assembly	2.1E-03	ACTN1/CTTN/SMAD3/THBS1/TLN1
GO:1902041	regulation of extrinsic apoptotic signaling pathway via death domain receptors	2.2E-03	BCL2L1/FGA/FGG/THBS1
GO:0070374	positive regulation of ERK1 and ERK2 cascade	2.2E-03	AGER/ARRB1/BRAF/CCL11/FGA/FGG/MAPK3
GO:0045807	positive regulation of endocytosis	2.2E-03	ANO6/ARRB1/CLU/LRP1/TF
GO:0050673	epithelial cell proliferation	2.2E-03	APOA1/BCL2L1/CCL11/IL6/PROK2/RUNX2/SMAD3/TBX1/TGFB2/THBS1
GO:0032368	regulation of lipid transport	2.2E-03	APOA1/CRH/ITGAV/LRP1/SPP1/THBS1
GO:2001236	regulation of extrinsic apoptotic signaling pathway	2.2E-03	BCL2L1/CTTN/FGA/FGG/ITGAV/THBS1
GO:0048661	positive regulation of smooth muscle cell proliferation	2.3E-03	CALCRL/IGF1R/IL6/MMP9/THBS1
GO:0010001	glial cell differentiation	2.3E-03	AGER/CLU/CSK/IL6/LRP1/MAPK3/VIM

ID	Description	p.adjust	geneID
GO:0051496	positive regulation of stress fiber assembly	2.4E-03	APOA1/BRAF/SMAD3/TPM1
GO:0010769	regulation of cell morphogenesis involved in differentiation	2.4E-03	APOA1/BRAF/FGA/FGG/P4HB
GO:0042063	gliogenesis	2.4E-03	AGER/CLU/CSK/IL6/LRP1/MAPK3/TGFB2/VIM
GO:0150115	cell-substrate junction organization	2.5E-03	ACTN1/CTTN/SMAD3/THBS1/TLN1
GO:0045778	positive regulation of ossification	2.6E-03	ADRB2/ANO6/SMAD3/TGFB2
GO:0061900	glial cell activation	2.6E-03	AGER/CLU/IL6/LRP1
GO:0070372	regulation of ERK1 and ERK2 cascade	2.6E-03	AGER/ARRB1/BRAF/CCL11/CSK/FGA/FGG/MAPK3
GO:2000630	positive regulation of miRNA metabolic process	3.0E-03	NR3C1/SMAD3/TGFB2/WT1
GO:0032963	collagen metabolic process	3.2E-03	IL6/MMP1/MMP13/MMP9/VIM
GO:0048143	astrocyte activation	3.4E-03	AGER/IL6/LRP1
GO:0030038	contractile actin filament bundle assembly	3.4E-03	APOA1/ARRB1/BRAF/SMAD3/TPM1
GO:0043149	stress fiber assembly	3.4E-03	APOA1/ARRB1/BRAF/SMAD3/TPM1
GO:0033002	muscle cell proliferation	3.5E-03	CALCRL/IGF1R/IL6/MMP9/TGFB2/THBS1/TPM1
GO:0031214	biomineral tissue development	3.6E-03	ADRB2/ANO6/MMP13/SMAD3/SPP1/TBX1
GO:0045926	negative regulation of growth	3.6E-03	ADRB2/BDKRB1/CDH1/SMAD3/SPP1/TGFB2/WT1
GO:0048660	regulation of smooth muscle cell proliferation	3.6E-03	CALCRL/IGF1R/IL6/MMP9/THBS1/TPM1
GO:1902893	regulation of miRNA transcription	3.9E-03	NR3C1/SMAD3/TGFB2/WT1
GO:0048659	smooth muscle cell proliferation	4.0E-03	CALCRL/IGF1R/IL6/MMP9/THBS1/TPM1
GO:0048771	tissue remodeling	4.0E-03	ADRB2/CSK/IL6/SPP1/TF/TIMP1
GO:0032233	positive regulation of actin filament bundle assembly	4.0E-03	APOA1/BRAF/SMAD3/TPM1
GO:0061614	miRNA transcription	4.0E-03	NR3C1/SMAD3/TGFB2/WT1

ID	Description	p.adjust	geneID
GO:1904036	negative regulation of epithelial cell apoptotic process	4.0E-03	BRAF/FGA/FGG/IGF1R
GO:0046885	regulation of hormone biosynthetic process	4.0E-03	ATP1A1/IGF1R/NR3C1
GO:0051235	maintenance of location	4.0E-03	APOA1/BDKRB1/FBN1/FTH1/HSP90B1/IL6/ITGAV/LTBP1
GO:0050708	regulation of protein secretion	4.0E-03	FGA/FGG/IL6/MYH10/SAA1/TGFB2/TTN
GO:0110148	biomineralization	4.0E-03	ADRB2/ANO6/MMP13/SMAD3/SPP1/TBX1
GO:0021782	glial cell development	4.3E-03	AGER/CLU/IL6/LRP1/VIM
GO:0030199	collagen fibril organization	4.3E-03	COL12A1/COL14A1/COLGALT1/TGFB2
GO:1903522	regulation of blood circulation	4.5E-03	AGER/ATP1A1/CHRM3/FGA/FGG/TGFB2/TPM1
GO:0030308	negative regulation of cell growth	4.8E-03	BDKRB1/CDH1/SMAD3/SPP1/TGFB2/WT1
GO:0031639	plasminogen activation	4.8E-03	FGA/FGG/THBS1
GO:0042730	fibrinolysis	4.8E-03	FGA/FGG/THBS1
GO:1903055	positive regulation of extracellular matrix organization	4.8E-03	COLGALT1/IL6/SMAD3
GO:0022612	gland morphogenesis	5.1E-03	CCL11/IL6/NR3C1/RXFP1/TGFB2
GO:0061448	connective tissue development	5.6E-03	MAPK3/MMP13/RUNX2/RXFP1/SMAD3/TIMP1/WT1
GO:0051495	positive regulation of cytoskeleton organization	5.6E-03	APOA1/BRAF/CCL11/CTTN/SMAD3/TPM1
GO:0030193	regulation of blood coagulation	5.7E-03	ANO6/FGA/FGG/THBS1
GO:0032103	positive regulation of response to external stimulus	5.9E-03	AGER/ANO6/BRAF/IGF1R/IL6/MAPK3/S100A12/SMAD3/THBS1
GO:1903532	positive regulation of secretion by cell	5.9E-03	CRH/FGA/FGG/MYH10/SPP1/TGFB2/TTN
GO:0002548	monocyte chemotaxis	5.9E-03	ANO6/CCL11/IL6/S100A12
GO:0050714	positive regulation of protein secretion	6.1E-03	FGA/FGG/MYH10/TGFB2/TTN
GO:0007271	synaptic transmission, cholinergic	6.1E-03	CHRM3/CHRNA1/LAMA2
GO:1900046	regulation of hemostasis	6.1E-03	ANO6/FGA/FGG/THBS1

ID	Description	p.adjust	geneID
GO:0032612	interleukin-1 production	6.2E-03	AGER/APOA1/IL6/SAA1/SMAD3
GO:0032652	regulation of interleukin-1 production	6.2E-03	AGER/APOA1/IL6/SAA1/SMAD3
GO:0060415	muscle tissue morphogenesis	6.3E-03	TBX1/TGFB2/TPM1/TTN
GO:1904888	cranial skeletal system development	6.3E-03	RUNX2/SMAD3/TBX1/TGFB2
GO:0033688	regulation of osteoblast proliferation	6.5E-03	IGF1R/ITGAV/SMAD3
GO:0048679	regulation of axon regeneration	6.5E-03	BRAF/IGF1R/SPP1
GO:1902042	negative regulation of extrinsic apoptotic signaling pathway via death domain receptors	6.5E-03	BCL2L1/FGA/FGG
GO:0032732	positive regulation of interleukin-1 production	6.5E-03	AGER/IL6/SAA1/SMAD3
GO:2000628	regulation of miRNA metabolic process	6.5E-03	NR3C1/SMAD3/TGFB2/WT1
GO:0007204	positive regulation of cytosolic calcium ion concentration	6.6E-03	BDKRB1/CXCR1/CXCR5/ITGAV/PROK2/SAA1
GO:0050818	regulation of coagulation	6.7E-03	ANO6/FGA/FGG/THBS1
GO:2000242	negative regulation of reproductive process	6.7E-03	BCL2L1/MYH9/TIMP1/WT1
GO:0010876	lipid localization	7.1E-03	ANO6/APOA1/CLU/CRH/IL6/ITGAV/LRP1/SPP1/THBS1
GO:0022411	cellular component disassembly	7.1E-03	ADAMTS4/ADRB2/CTTN/IGF1R/IL6/LRP1/MMP1/MMP13/MMP9
GO:0030100	regulation of endocytosis	7.1E-03	ANO6/ARRB1/CLU/ITGAV/LRP1/TF
GO:1902903	regulation of supramolecular fiber organization	7.1E-03	APOA1/BRAF/CCL11/CLU/COLGALT1/CTTN/SMAD3/TPM1
GO:0061041	regulation of wound healing	7.1E-03	ANO6/FGA/FGG/SMAD3/THBS1
GO:1903036	positive regulation of response to wounding	7.1E-03	ANO6/BRAF/IGF1R/THBS1

ID	Description	p.adjust	geneID
GO:0051222	positive regulation of protein transport	7.2E-03	CDH1/FGA/FGG/MYH10/SMAD3/TGFB2/TTN
GO:0061045	negative regulation of wound healing	7.4E-03	FGA/FGG/SMAD3/THBS1
GO:0001667	ameboidal-type cell migration	7.8E-03	APOA1/BRAF/MMP9/MYH9/S100P/TBX1/TGFB2/THBS1/TIMP1
GO:0070570	regulation of neuron projection regeneration	7.9E-03	BRAF/IGF1R/SPP1
GO:0048644	muscle organ morphogenesis	8.0E-03	TBX1/TGFB2/TPM1/TTN
GO:2000352	negative regulation of endothelial cell apoptotic process	8.5E-03	BRAF/FGA/FGG
GO:0051047	positive regulation of secretion	8.5E-03	CRH/FGA/FGG/MYH10/SPP1/TGFB2/TTN
GO:0033687	osteoblast proliferation	9.1E-03	IGF1R/ITGAV/SMAD3
GO:0035909	aorta morphogenesis	9.1E-03	LRP1/TBX1/TGFB2
GO:0051592	response to calcium ion	9.3E-03	BRAF/FGA/FGG/THBS1/TTN
GO:0010921	regulation of phosphatase activity	9.3E-03	HSP90B1/ITGA1/SMAD3/TGFB2
GO:1904951	positive regulation of establishment of protein localization	9.3E-03	CDH1/FGA/FGG/MYH10/SMAD3/TGFB2/TTN
GO:0032970	regulation of actin filament-based process	9.4E-03	APOA1/BRAF/CCL11/CTTN/LRP1/MYH9/SMAD3/TPM1
GO:0008625	extrinsic apoptotic signaling pathway via death domain receptors	9.6E-03	BCL2L1/FGA/FGG/THBS1
GO:0034103	regulation of tissue remodeling	9.6E-03	CSK/IL6/SPP1/TF
GO:0042692	muscle cell differentiation	9.7E-03	ACTN1/FHL2/LAMA2/MYH9/TBX1/TPM1/TTN/WT1
GO:0006909	phagocytosis	9.8E-03	ANO6/APOA1/CSK/ITGAV/LRP1/MYH9/THBS1

Table S7: 196 significant pathways with adjusted p-value < 0.01 based on GO enrichment analysis

Table S8. Number of drugs targeting the tier 1 druggable genes

gene	Druggable Tier	NumDrug	Drugs or biomedical entities targeting the genes
**ADRB2**	Tier_1	81	Spermine; Amphetamine; Betaxolol; Bethanidine; Isoetarine; Cabergoline; Metoprolol; Amitriptyline; Olanzapine; Atenolol; Norepinephrine; Mirtazapine; Timolol; Phenylpropanolamine; Dipivefrin; Sotalol; Carteolol; Nortriptyline; Propranolol; Labetalol; Bisoprolol; Epinephrine; Trimipramine; Orciprenaline; Dobutamine; Pseudoephedrine; Alprenolol; Ritodrine; Terbutaline; Bitolterol; Phenoxybenzamine; Salmeterol; Pindolol; Formoterol; Salbutamol; Ergoloid mesylate; Isoprenaline; Arbutamine; Carvedilol; Desipramine; Propafenone; Acebutolol; Nadolol; Levobunolol; Metipranolol; Arformoterol; Fenoterol; Pirbuterol; Bevantolol; Penbutolol; Ephedra; Mephentermine; Procaterol; Clenbuterol; Bambuterol; Oxprenolol; Putrescine; Spermidine; Celiprolol; Nebivolol; Indacaterol; NCX 950; Asenapine; Droxidopa; Bufuralol; Protokylol; (2S)-1-(9H-Carbazol-4-yloxy)-3-(isopropylamino)propan-2-ol; Bopindolol; Bupranolol; Befunolol; Olodaterol; Vilanterol; Arotinolol; Doxofylline; Racepinephrine; Dihydroergocornine; DL-Methylephedrine; Etafedrine; Tulobuterol; Levosalbutamol; Dihydroergocristine
**ATP1A1**	Tier_1	27	Digoxin; Acetyldigitoxin; Hydroflumethiazide; Etacrynic acid; Trichlormethiazide; Deslanoside; Ouabain; Diazoxide; Bretylium; Ciclopirox; Bepridil; Potassium cation; Aluminium; Magnesium cation; Digitoxin; Almitrine; Istaroxime; Rubidium Rb-82; Potassium gluconate; Magnesium gluconate; Magnesium acetate; Potassium acetate; Potassium sulfate; Magnesium levulinate; Magnesium lactate; Aluminium phosphate; Aluminum acetate
**BCL2L1**	Tier_1	3	4'-FLUORO-1,1'-BIPHENYL-4-CARBOXYLIC ACID; Isosorbide; Gossypol
**BDKRB1**	Tier_1	6	Ramipril; Captopril; Zinc; Enalaprilat; Zinc acetate; Zinc chloride
**BRAF**	Tier_1	11	Sorafenib; XL281; RAF-265; N-{3-[(5-chloro-1H-pyrrolo[2,3-b]pyridin-3-yl)carbonyl]-2,4-difluorophenyl}propane-1-sulfonamide; N-{2,4-difluoro-3-[(5-pyridin-3-yl-1H-pyrrolo[2,3-b]pyridin-3-yl)carbonyl]phenyl}ethanesulfonamide; (1E)-5-(1-piperidin-4-yl-3-pyridin-4-yl-1H-pyrazol-4-yl)-2,3-dihydro-1H-inden-1-one oxime; Vemurafenib; Regorafenib; Dabrafenib; Encorafenib; Fostamatinib
**CALCRL**	Tier_1	3	Olcegepant; Telcagepant; Erenumab
**CCL11**	Tier_1	1	CAT-213
**CHRM3**	Tier_1	84	Cevimeline; Tramadol; Succinylcholine; Ziprasidone; Disopyramide; Amitriptyline; Ipratropium; Olanzapine; Metixene; Terfenadine; Clozapine; Trihexyphenidyl; Oxyphencyclimine; Procyclidine; Loxapine; Carbamoylcholine; Promazine; Hyoscyamine; Cyproheptadine; Meperidine; Imipramine; Methscopolamine bromide; Chlorpromazine; Darifenacin; Tridihexethyl; Anisotropine methylbromide; Nortriptyline; Amoxapine; Lamotrigine; Cinnarizine; Atropine; Thiopental; Nicardipine;

			Paroxetine; Homatropine Methylbromide; Trimipramine; Diphemanil Methylsulfate; Scopolamine; Benzquinamide; Propiomazine; Cryptenamine; Tropicamide; Brompheniramine; Maprotiline; Glycopyrronium; Bethanechol; Tolterodine; Oxybutynin; Promethazine; Pilocarpine; Doxepin; Desipramine; Ketamine; Quetiapine; Mivacurium; Diphenidol; Aripiprazole; Chlorprothixene; Pancuronium; Pipecuronium; Methotrimeprazine; Tiotropium; Solifenacin; Isopropamide; Acetylcholine; Arecoline; Mepenzolate; ALKS 27; Pizotifen; Fesoterodine; Methacholine; Hexocyclium; Aclidinium; Umeclidinium; Trimebutine; Dosulepin; Etoperidone; Imidafenacin; Butylscopolamine; Homatropine; Thonzylamine; Revefenacin; Propiverine; Aripiprazole lauroxil
**CHRNA1**	Tier_1	2	Galantamine; Agmatine
**CSK**	Tier_1	4	Dasatinib; Staurosporine; TG-100801; Fostamatinib
**CTSD**	Tier_1	7	Insulin Human; Insulin Pork; S-Methylcysteine; 1h-Benoximidazole-2-Carboxylic Acid; N-Aminoethylmorpholine; 5-AMINO-6-CYCLOHEXYL-4-HYDROXY-2-ISOBUTYL-HEXANOIC ACID; CYCLOHEXYLMETHYL-2,3-DIHYDROXY-5-METHYL-HEXYLAMIDE
**CXCR1**	Tier_1	1	Ketoprofen
**FGA**	Tier_1	17	Alteplase; Reteplase; Anistreplase; Tenecteplase; Sucralfate; Zinc; Alfimeprase; Ancrod; EP-2104R; Lanoteplase; Thrombin; Prothrombin; Human Thrombin; Thrombin Alfa; Anti-inhibitor coagulant complex; Zinc acetate; Zinc chloride
**FGG**	Tier_1	4	Sucralfate; Thrombin; Human Thrombin; Thrombin Alfa
**IGF1R**	Tier_1	16	Insulin Human; Insulin Lispro; Insulin Glargine; Insulin Pork; Mecasermin; Phosphoaminophosphonic Acid-Adenylate Ester; ATL1101; XL228; rhIGFBP-3; Linsitinib; Teprotumumab; (4Z)-6-bromo-4-({[4-(pyrrolidin-1-ylmethyl)phenyl]amino}methylidene)isoquinoline-1,3(2H,4H)-dione; 3-[5-(1H-IMIDAZOL-1-YL)-7-METHYL-1H-BENZIMIDAZOL-2-YL]-4-[(PYRIDIN-2-YLMETHYL)AMINO]PYRIDIN-2(1H)-ONE; Somatrem; Cixutumumab; Brigatinib
**IL6**	Tier_1	13	Ginseng; YSIL6; VX-702; Atiprimod; CRx-139; Andrographolide; Tapinarof; Siltuximab; Polaprezinc; Foreskin fibroblast (neonatal); Foreskin keratinocyte (neonatal); Binimetinib; Dilmapimod
**ITGAV**	Tier_1	1	Antithymocyte immunoglobulin (rabbit)
**MAPK3**	Tier_1	7	Sulindac; Arsenic trioxide; Purvalanol; 5-iodotubercidin; Seliciclib; Cholecystokinin; Ulixertinib
**MGAM**	Tier_1	3	Acarbose; Miglitol; Voglibose
**MMP1**	Tier_1	6	Marimastat; N-HYDROXY-2(R)-[[(4-METHOXYPHENYL)SULFONYL](3-PICOLYL)AMINO]-3-METHYLBUTANAMIDE HYDROCHLORIDE; N-[3-(N'-HYDROXYCARBOXAMIDO)-2-(2-METHYLPROPYL)-PROPANOYL]-O-TYROSINE-N-METHYLAMIDE; METHYLAMINO-PHENYLALANYL-LEUCYL-HYDROXAMIC ACID; [[1-[N-HYDROXY-ACETAMIDYL]-3-METHYL-BUTYL]-CARBONYL-LEUCINYL]-ALANINE ETHYL ESTER; N-HYDROXY-2-[4-(4-PHENOXY-BENZENESULFONYL)-TETRAHYDRO-PYRAN-4-YL]-ACETAMIDE

**MMP13**	Tier_1	14	Marimastat; 3-Methylpyridine; 2-{4-[4-(4-Chloro-Phenoxy)-Benzenesulfonyl]-Tetrahydro-Pyran-4-Yl}-N-Hydroxy-Acetamide; WAY-151693; Hydroxyaminovaline; 1-Methyloxy-4-Sulfone-Benzene; PYRIMIDINE-4,6-DICARBOXYLIC ACID BIS-(3-METHYL-BENZYLAMIDE); PYRIMIDINE-4,6-DICARBOXYLIC ACID BIS-(4-FLUORO-3-METHYL-BENZYLAMIDE); PYRIMIDINE-4,6-DICARBOXYLIC ACID BIS-[(PYRIDIN-3-YLMETHYL)-AMIDE]; TERT-BUTYL 4-({[4-(BUT-2-YN-1-YLAMINO)PHENYL]SULFONYL}METHYL)-4-[(HYDROXYAMINO)CARBONYL]PIPERIDINE-1-CARBOXYLATE; 4-{[1-METHYL-2,4-DIOXO-6-(3-PHENYLPROP-1-YN-1-YL)-1,4-DIHYDROQUINAZOLIN-3(2H)-YL]METHYL}BENZOIC ACID; 5-(2-ETHOXYETHYL)-5-[4-(4-FLUOROPHENOXY)PHENOXY]PYRIMIDINE-2,4,6(1H,3H,5H)-TRIONE; CTS-1027; BENZYL 6-BENZYL-5,7-DIOXO-6,7-DIHYDRO-5H-[1,3]THIAZOLO[3,2-C]PYRIMIDINE-2-CARBOXYLATE
**MMP9**	Tier_1	15	Glutathione; Marimastat; Minocycline; Captopril; Glucosamine; Zinc; 2-Amino-N,3,3-Trimethylbutanamide; 2-{[Formyl(Hydroxy)Amino]Methyl}-4-Methylpentanoic Acid; AE-941; PG-530742; 5-(4-PHENOXYPHENYL)-5-(4-PYRIMIDIN-2-YLPIPERAZIN-1-YL)PYRIMIDINE-2,4,6(2H,3H)-TRIONE; (2R)-2-AMINO-3,3,3-TRIFLUORO-N-HYDROXY-2-{[(4-PHENOXYPHENYL)SULFONYL]METHYL}PROPANAMIDE; (3R)-4,4-DIFLUORO-3-[(4-METHOXYPHENYL)SULFONYL]BUTANOIC ACID; Zinc acetate; Zinc chloride
**NR3C1**	Tier_1	60	Flunisolide; Diflorasone; Alclometasone; Medrysone; Amcinonide; Fluorometholone; Megestrol acetate; Beclomethasone dipropionate; Progesterone; Spironolactone; Betamethasone; Desoximetasone; Fluticasone propionate; Fluocinolone acetonide; Ulobetasol; Triamcinolone; Prednisone; Flumethasone; Fludrocortisone; Hydrocortisone; Mometasone; Hydrocortamate; Mifepristone; Clocortolone; Flurandrenolide; Prednisolone; Loteprednol etabonate; Rimexolone; Methylprednisolone; Clobetasol propionate; Fluocinonide; Prednicarbate; Fluoxymesterone; Budesonide; Dexamethasone; Desonide; Cortisone acetate; Paramethasone; Ciclesonide; Hexane-1,6-Diol; Aldosterone; ORG-34517; Difluprednate; Ulipristal; Fluticasone furoate; Tixocortol; Difluocortolone; Gestrinone; Onapristone; Cortivazol; Clobetasone; Mometasone furoate; Hydrocortisone aceponate; Hydrocortisone acetate; Hydrocortisone butyrate; Hydrocortisone cypionate; Hydrocortisone phosphate; Hydrocortisone probutate; Hydrocortisone valerate; Segesterone acetate
**PPIB**	Tier_1	2	Proline; 1,4-Dithiothreitol
**SLC2A5**	Tier_1	3	D-glucose; Invert sugar; Fludeoxyglucose F-18
**VIM**	Tier_1	2	Artenimol; Phenethyl Isothiocyanate

Table S8: Number of drugs targeting the tier 1 druggable genes

2 Supplement Result

2.1 Validation of GOLD IV DEGs

In order to validate the 168 differentially expressed genes (DEGs) identified in the discovery dataset, we compared this gene list to the DEGs identified in a validation dataset with similar COPD cohort (GSE76925, Table S9). Although different microarray platforms were used in the two datasets, 12,864 common genes were screened by both platforms in the discovery and validation dataset. In the discovery dataset, 139 out of the 168 DEGs belong to the commonly screened genes. In the validation dataset, 528 genes were identified as DEGs in the GOLD IV patients by the same analysis approach in the discovery dataset. There are 34 overlapping DEGs between the discovery and validation sets (Figure S1). Moreover, the fold change directions of most the overlapping DEGs are consistent (Table S10).

Table S9. Sample demographics of discovery and validation transcriptomics sets

	**Discovery set**		**Validation set**	
	**GOLD IV**	**Control**	**GOLD IV**	**Control**
Totals	54	65	71	40
Age (years, mean ± SD)	57.2 ± 8.4	65.6 ± 10.4	61.9 ± 5.6	65.7 ± 9
Gender (male/female)	24/30	36/29	37/34	15/25
Smoking status (non/ex-smokers)	1/53	2/63	0/71	0/40

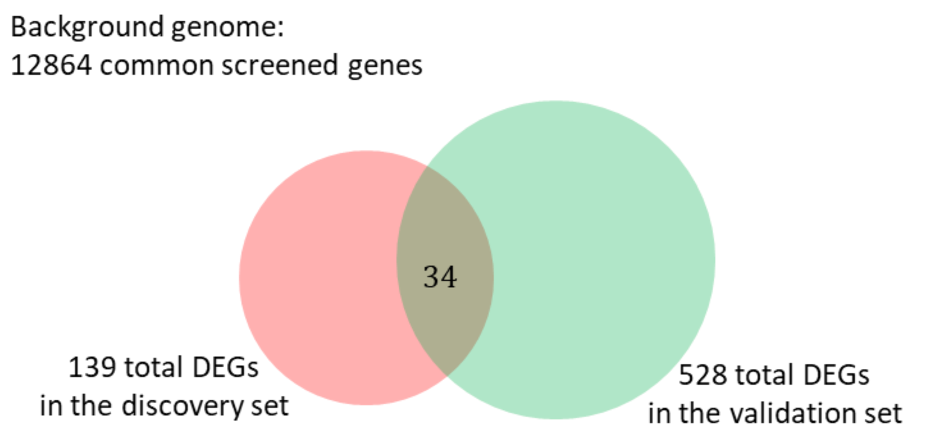

Fisher's exact test (p < 2.2e-16)

	DEGs in discovery set	Genome
DEGs in validation set	34	528
DEGs not in validation set	139 – 34 = 105	12864 – 528 = 12336

DEGs: differentially expressed genes

Figure S1. Differentially expressed genes identified by discovery and validation sets. In the validation dataset, 528 genes were identified as DEGs in the GOLD IV patients by the same analysis approach in the discovery dataset. There are 34 overlapping DEGs between the discovery and validation sets. We statistically evaluated whether the ratio of 34/139 overlapping genes would appear due to random chance by comparing with the ratio of 528 validation DEGs in the 12,864 background genome. The over-representation test reported a p-value $< 10^{-15}$ based on the Fisher exact test on the contingency table, indicating that our DEGs identified in the discovery set was also associated with the DEGs in the validation set.

	discovery set		validation set		comparison
**GENE_SYMBOL**	**logFC**	**adj.P.Val**	**logFC**	**adj.P.Val**	**foldChangeDirection**
BHLHE22	1.408752	5.24E-12	1.174028	1.65E-05	Same
CA3	1.633705	3.26E-10	1.792772	2.35E-05	Same
TIMP4	1.585092	1.16E-09	1.288547	3.06E-04	Same
CCL19	1.92926	7.12E-09	1.609784	2.05E-04	Same
COL10A1	1.822596	8.26E-09	1.555302	1.92E-03	Same
SLC1A3	1.127254	1.99E-08	1.103234	1.42E-04	Same
PLA2G7	1.59356	2.3E-08	1.25119	4.52E-04	Same
CXCR5	1.783988	2.44E-08	-1.59364	1.34E-03	not same
LEPR	-1.06663	4.24E-08	-1.30409	3.44E-03	Same
MMP9	1.567832	7.06E-08	-1.32956	2.02E-03	not same
BLK	1.720591	8.32E-08	1.558954	7.93E-05	Same
CD19	1.756526	8.92E-08	1.988151	5.39E-05	Same
FCRLA	2.094148	1.74E-07	1.807438	1.62E-04	Same
COMP	1.786859	1.75E-07	1.583639	1.29E-03	Same
HS3ST2	1.428245	2.17E-07	1.372918	5.35E-04	Same
DPYS	1.318612	2.62E-07	1.759433	1.33E-05	Same
POU2AF1	1.552449	5E-07	1.304951	5.61E-04	Same
DAPL1	1.83263	7.47E-07	1.412726	3.05E-03	Same
GREM1	1.938453	1.86E-06	1.380173	1.74E-03	Same
CHRNA1	1.786101	2.05E-06	-1.45168	7.78E-04	not same
SLITRK6	-1.3683	3.66E-06	-1.86924	9.74E-04	Same
TM4SF19	1.423956	9.67E-06	-1.81713	1.53E-04	not same
SPP1	1.886012	1.37E-05	1.529668	3.22E-04	Same
SAA1	1.917719	2.63E-05	-1.97729	1.31E-03	not same
FGG	2.122791	6.01E-05	1.692871	8.39E-04	Same
CXCL13	2.277401	6.05E-05	1.827646	2.00E-03	Same
MMP11	1.201676	9.36E-05	1.659526	2.12E-04	Same
TNFRSF13B	1.022567	9.39E-05	1.273341	1.12E-03	Same
CHIT1	1.925543	0.000129	-1.44891	4.30E-03	not same
SSTR1	-1.02871	0.000275	-1.3739	1.38E-03	Same
RPRML	1.229686	0.000287	1.157391	8.48E-03	Same
KRT17	1.778762	0.000529	1.238003	1.81E-02	Same
BIRC7	1.088267	0.000729	1.082787	3.32E-03	Same
FGA	1.732555	0.000988	1.064202	3.88E-02	Same

Table S10. DEGs between GOLD IV vs healthy control in both discovery and validation set

Next, we statistically evaluated whether the ratio of 34/139 overlapping genes would appear due to random chance by comparing with the ratio of 528 validation DEGs in the 12,864 background genome. The over-representation test reported a p-value < 10^{-15} based on the Fisher exact test on the contingency table in (Figure S1), indicating that our DEGs identified in the discovery set was also associated with the DEGs in the validation set.

In addition, we further validate our DEGs with a second approach of gene set enrichment analysis. Both the up-regulated and down-regulated DEGs in the discovery set were found to be significantly enriched in the validation COPD cohort when they were compared to the genes of the validation set ranked by differential expression analysis p-values (Table S11). As shown in the enrichment plot, genes in the validation set were ranked by the signed log-transformed p-values. Hence, genes most significantly up-regulated will be at the top of the ranked list, and genes most significantly down-regulated will be at the bottom of the ranked list (Figure S2). GSEA found that up-regulated genes in the discovery set showed up more frequently at the top of the ranked validation gene list. Similarly, the down-regulated genes in the discovery set located more frequently at the bottom of the ranked validation gene list. This suggested an enrichment of both the up-regulated and down-regulated genes in the validation set. On the other hand, the negative control of genes up-regulated in Alzheimer's disease was not shown to be enriched in the validation set.

**Gene set**	**P-value**	**Adjusted p-value**	**Enrichment Score**	**Normalized Enrichment Score**	**No. of overlapping genes**

DEG_Discovery_up	0.00116	0.00349	0.63554	2.13579	106
DEG_Discovery_down	0.00437	0.00655	-0.70377	-2.2467	31
Control_Alzheimer_up	0.94206	0.94206	0.23381	0.87969	1228

Table S11. Gene set enrichment analysis result table for DEGs in the discovery set

Enrichment Plot: down-regulated DEGs in discovery set

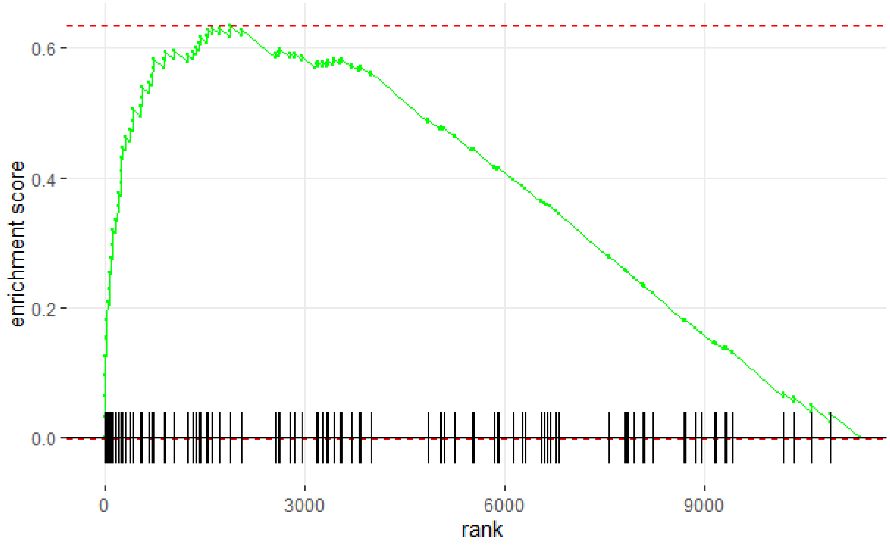

Enrichment Plot: up-regulated DEGs in discovery set

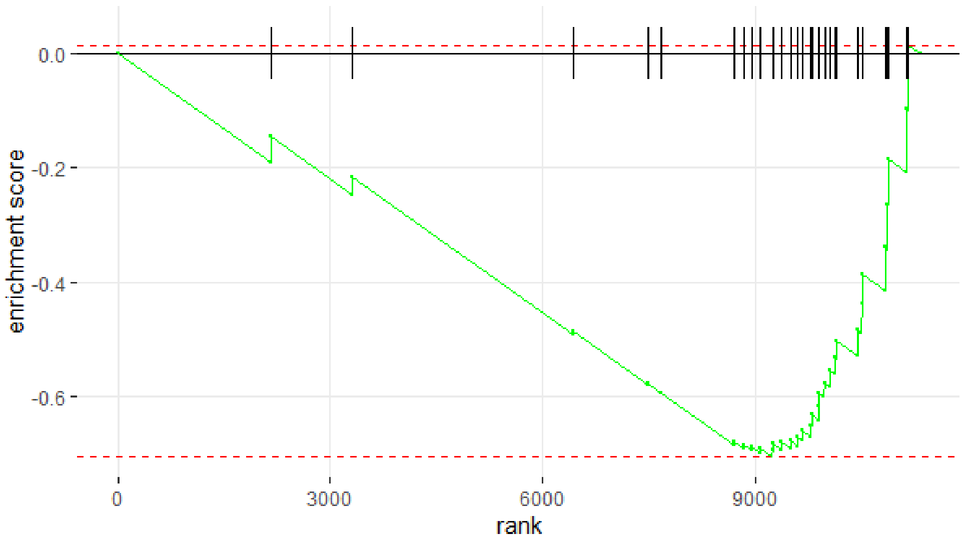

Figure S2. Gene set enrichment analysis plot for up-regulated and down-regulated DEGs in the discovery set.

Rank: after differential gene expression analysis, genes in the validation set are ranked in decreasing order by –log10(p-values) * sign of the fold change. **Black vertical line**: gene set hits - DEGs in the discovery set are found in the validation set. **Green line**: Enrichment score profile - when there is a hit, the enrichment score will increase and the green line will climb up. Otherwise, the enrichment score will decrease and the green line will go down. **Red dotted line**: max and min enrichment score.

Besides the gene level comparison, we also evaluate the similarity of the DEGs between the discovery and the validation dataset on the pathway level. Gene Ontology enrichment analysis on the 528 DEGs from the validation dataset revealed 33 same over-represented biological processes in GOLD IV patients, including leukocyte activation (GO:0045321), cellular response to cytokine stimulus (GO:0071345) and positive regulation of cell-cell adhesion (GO:0022409).

Overall, compared with a similar COPD transcriptomics dataset, our DEGs were well supported by two different validation approaches. The over-representation test and GSEA are widely used in the bioinformatics fields as approaches for pathway enrichment analysis. Here, we employed these two approaches for the validation of DEGs identified in the discovery set, as we can readily extend the use of the two methods when we treated our DEGs as a pathway or a gene set related to COPD. The same approach has been used by another published study to compare their DEGs to other transcriptomic datasets [53]. However, there are still some limitations to such validation approaches. The percentage of DEGs detected by both discovery and validation set was not high. A similar inconsistency was also observed in another COPD transcriptomic study. Several factors may contribute to this. The first reason was the biological difference among subjects. Even though we have limited our analysis to GOLD IV patients to control the disease heterogeneity, the patients may still represent different subgroups of the disease in terms of other pathophysiological status related to COPD. The second reason is the sample size. Despite that this is already the largest COPD transcriptomics dataset with the necessary disease clinical information for re-analysis, thousands of samples may be needed to obtain a more consistent list of DEGs due to the heterogeneous nature of the disease and small variations on the expression level changes. The third reason is the different platforms. The

discovery set was based on the Agilent platform and the validation set was based on the Illumina platform, and cross-platform variation was shown to exist for the expression pattern of some genes [54]. Nevertheless, it has been demonstrated in a microarray reproducibility study that most of the DEGs identified in a microarray study were still likely true positives even the percent of overlapping genes with other studies appeared to be low [21]. Therefore, we moved forward with the DEGs in the discovery set for further analysis in the pipeline.

3 Material and Method

3.1 Transcriptomic data

We obtained COPD lung transcriptomics datasets for the identification of initial COPD signature genes from publicly available sources. We searched for transcriptomics data generated by microarray and RNA-Sequencing experiments. The search was performed on the Gene Expression Omnibus (GEO) database (www.ncbi.nlm.nih.gov/geo/) [55].

Specifically, we queried the GEO database with the keyword “COPD” and “Homo sapiens” and screened only for GEO Series datasets generated on lung tissues. Datasets without COPD status and healthy control samples were excluded. Finally, the dataset with the greatest number of human samples across COPD GOLD stages was used as the discovery dataset for the identification of initial signature genes in COPD. COPD subjects from GOLD 0 group or without GOLD stage level, demographic or smoking status were excluded from the analysis. One additional dataset with subjects of similar COPD stages (based on the COPD GOLD classification) was included as the validation dataset.

All datasets were downloaded within R by the GEOquery package [56]. The subjects with missing gender, age, or smoking status were excluded. The comparison of gender and smoking status between groups was performed using Fisher’s exact test. The difference in the age between groups was analyzed with one way ANOVA test followed by pairwise t tst.

3.2 Differential expression analysis

For the differential expression analysis, we used the limma package [45]. It fits the gene expression to a linear model and used empirical Bayes to borrow information between genes in a dynamic way. Age, gender of the subjects and sample batch were used in the linear model as covariates. Then t-statistics was applied to identify the up-regulated and down-regulated probes in GOLD IV patients vs. healthy controls. In order to adjust for the random appearance of significant results during multiple statistics analysis, adjusted p-values were obtained by applying Benjamini and Hochberg multiple comparison adjustment to the raw p-values [57]. The probe IDs were mapped to gene symbols with the Agilent chip annotation package "hgug4112a.db" in R [58]. Finally, COPD signature genes were selected based on both adjusted p-values and fold change of the gene expression levels. The differential gene analysis results were visualized as a volcano plot.

3.3 Functional enrichment

We performed over-representation analysis on the DEGs from differential expression analysis. Enriched biological processes in COPD were identified with the pathway annotations in the gene ontology database (http://geneontology.org). This analysis was carried out by DOSE package in R and visualized with clusterProfiler package [46,47].

3.4 Gene set enrichment analysis

Gene set enrichment analysis (GSEA) was performed to evaluate whether the discovery set DEGs were enriched in the validation COPD cohort. GSEA algorithm compares gene sets to a ranked gene list and calculates enrichment score and p-values by permutation test [49]. First, a ranked gene list was constructed by sorting genes in the validation set by the signed p-values from differential expression analysis (equation 1) in decreasing order. Next, differentially expressed genes (DEGs) in the discovery set were split into two gene sets of up-regulated and down-regulated DEGs. A negative control gene set of genes up-regulated in Alzheimer's disease was obtained from GSEA Molecular Signatures Database [59,60]. The selection of the background genome may affect the performance of GSEA by sample source bias[61]. Therefore, we selected the genes that were detected in both discovery and validation sets as background genome. The ranked gene list and gene sets were updated to only include genes from the background genome. Finally, the GSEA analysis and visualization were carried out by the fgsea package in R [62].

$$\text{Ranking metrics} = sign(log_2\text{fold\_change}) \times -log_{10}(\text{p\_value})$$

Equation E1. Determination of ranking metrics

3.5 Over-representation test of the differentially expressed genes

The over-representation test was performed to assess whether the differentially expressed genes (DEGs) identified in the discovery set were associated with the DEGs identified in the validation set. In principle, the ratio of the overlapping DEGs is compared to the random chance

that such overlap may happen to another gene set in the genome (DEGs in the validation set in this case) with hypergeometric distribution by fisher exact test [48]. In order to properly run the over-representation test, we first defined a uniform background genome to address the inconsistent numbers of probe genes in the microarray platforms in the discovery and validation set. The set of genes screened by both microarray platforms was used as the background genome. Next, we identified DEGs in GOLD IV compared to healthy controls in the validation set with the same age-and-gender-adjusted linear model as in the discovery set. Then we updated both discovery and validation DEGs by only including the ones in the previously defined background genome. Finally, the Fisher exact test was performed on the 2 x 2 contingency table with the number of overlapping DEGs, the number of remaining non-overlapping DEGs in the discovery set, the number of DEGs in the validation datasets, the number of genes not identified as validation DEGs in the genome (Table S12).

Table S12. Contingency table for over-representation test

	DEGs in discovery set (D)	Genome (G)
DEGs in validation set (V)	$D \cap V$	$G \cap V$
DEGs not in validation set	$D - D \cap V$	$G - G \cap V$

DEGs: Differentially expressed genes. D: DEGs in discovery set. V: DEGs in validation set. G: Common genes screened by both discovery and validation set

3.6 GWAS data

GWAS related to COPD was identified through PubMed search with the keyword "COPD AND ('genome-wide association study')". The abstracts and material sections were reviewed to filter out unrelated publications. In addition, publications mentioned in the COPD GWAS meta-analysis were reviewed to complement the GWAS literature that may be missed during the PubMed search. Information on the subject disease status, genetic loci associated with COPD, and susceptible genes were obtained from GWAS literature.

3.7 Proteomics data

A list of differentially expressed proteins in lung tissues of COPD patients was obtained from COPD proteomics literature. The literature search was done by searching the keyword "(COPD lung) AND (proteome OR proteomics)" on the PubMed website (pubmed.ncbi.nlm.nih.gov). Then the abstracts and the material sections of the papers in the search results were checked for the information on the disease status of subjects, the tissue source, and the experimental approaches on sample analysis. Finally, a list of differentially expressed proteins was compiled based on the results of the papers containing proteomics analysis on lung tissues from COPD patients. The protein names were matched to corresponding gene symbols by searching on the UniProt website (www.uniprot.com) for the purpose of multi-omics integration.

3.8 Metabolomics data

Metabolomics study related to COPD was identified through PubMed search with the keyword "(COPD lung) AND (metabolomics OR metabolome)". Information on the subject disease status, sample source, and experimental approaches were obtained from the abstracts and the material sections of the papers in the search results. Metabolomics studies based on bronchoalveolar lavage fluid from COPD patients were included as the source for the identification of metabolites associated with COPD. Metabolomics class information was obtained from the Metabolomics Workbench (www.metabolomicsworkbench.org).

3.9 Integration of metabolomics data with MAGI

The MAGI (metabolome and genome integration) software was used to identify proteins and reactions linked to the metabolites associated with COPD [50]. The metabolites compound names were mapped to the IUPAC International Chemical Identifier (InChI) keys as MAGI inputs [63]. MAGI score of greater than 4 was used as criteria to filter proteins with a high confidence level of the metabolites and genes association. Protein names were mapped to gene symbols for the uniform integration with other omics levels.

3.10 Protein-protein interaction data

We obtained the physical and functional protein-protein interaction data from the STRING database v11 [51]. The interactions were based on several types of sources, including experimental data, computational prediction, and public literature text mining. We filtered for

interactions with high confidence level interactions (interaction score >=0.7) to be included in the analysis.

3.11 Calculation of distances between candidate genes and omics signature genes

The distances between genes were calculated from the protein-protein interaction network. Figure S3 is an illustration of the distances between a candidate gene and two other genes in the network, as well as the distance of the candidate gene to itself.

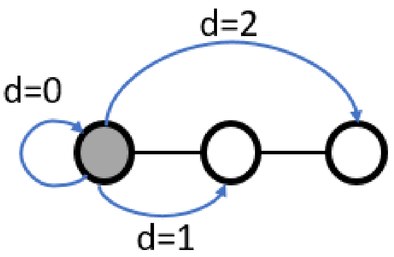

Figure S3. Illustration of distances between genes in the network

Gray circle: candidate gene; white circle: additional genes in the network; black line: physical or functional interaction between two genes; blue line: distances of candidate genes to genes in the network

First, three distances matrices were created from the paired protein interaction table retrieved from STRING database: 1) distance between COPD genomics signature genes and all candidate genes in the genome, 2) distance between COPD transcriptomics signature genes and all candidate genes in the genome, and 3) distance from all candidate genes in the genome to COPD signature genes identified from proteomics study or proteins linked to COPD

metabolomics. The distance matrices were created with the igraph package and visualized with the pheatmap package in R.

Next, the shortest distances of an individual candidate gene to three sets of omics signature genes were calculated as $d^{Transcriptomics}$, d^{GWAS}, and $d^{Protein}$, as shown in the example in Figure S4. For each candidate gene, it had multiple distance values representing distances to all genes in the signature gene set. The shortest distance was used to represent the proximity of the candidate gene to the individual omics signature gene set.

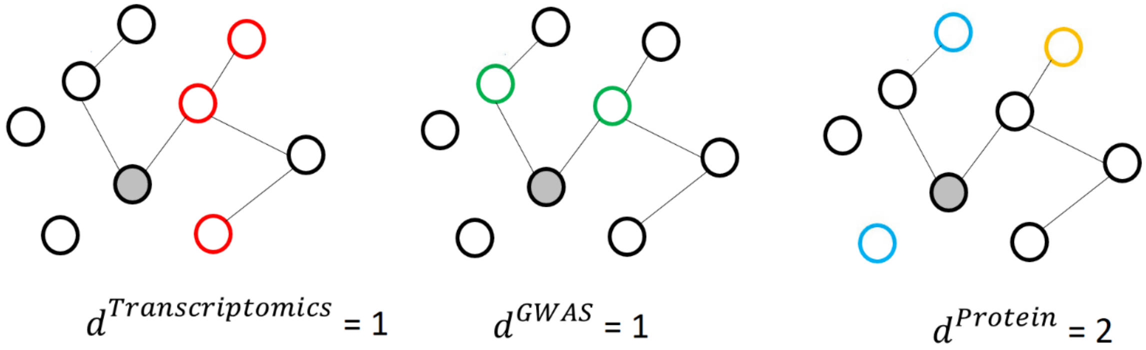

Figure S4. Shortest distances of a candidate gene to three sets of omics signature genes

Circle: genes; grey circle: candidate gene; red circle: COPD genomics signature genes; green circle: COPD transcriptomics signature genes; blue circle: COPD proteomics signature genes; yellow circle: proteins linked to the COPD-associated metabolomics

Finally, $d^{Transcriptomics}$, d^{GWAS}, and $d^{Protein}$ were summed up to a sum distance to represent the overall proximity of a candidate gene to all omics signature genes (Equation E2). A smaller sum distance indicates a closer association to signature genes from all omics levels.

$$\hat{d}_i^{sum} = \min_j d_{ij}^{Transcriptomics} + \min_j d_{ij}^{GWAS} + \min_j d_{ij}^{Protein}$$

Equation E2. Determination of sum distance as overall proximity to all omics signature genes

*i is the candidate gene and j is the gene to calculate distance from in the network

3.12 Heat diffusion network analysis with Cytoscape

Cytoscape 3.7.0 [64], a network analysis software, was used for the implementation of the heat diffusion network analysis. First, the whole human interactome was retrieved from the STRING database through the EnrichmentMap app 3.1.0 in Cytoscape. Only interactions with greater than 0.7 STRING interaction scores were loaded into the software. Next, the signature genes identified from genomics, transcriptomics, proteomics, metabolomics levels were imported into Cytoscape as gene node annotation table. Then, heat diffusion network analysis was carried out with the diffusion plugin 1.6.0 in Cytoscape [24]. The nodes representing signature genes from all omics levels were selected as diffusion network input, and the analysis was run with the default settings.

3.13 The retrieval of drug-gene target information

The drugs targeting the druggable genes were found on the DrugBank database (version 5.1.4, released 2019-07-02). This database is a well-organized resource that holds both comprehensive drug information and drug-target data [65]. Specifically, drug-gene pairs were obtained by parsing the xml database files with the XML package in R.

3.14 The automated literature search for COPD studies with repurposing drugs

The literature search was carried out to find supportive information on drug repurposing opportunities. The PubMed search was performed on batch with an R script and NCBI API. For each drug targeting the candidate genes, the number of literatures found in PubMed search by keyword “COPD + drug” was obtained. This number was used for the preliminarily screening of drug relevance to COPD. A manual literature search was carried out for the follow-up with the drugs that have been involved in COPD studies.

3.15 Connectivity map analysis

The COPD transcriptomic profiles obtained from the previous differential gene expression analysis were analyzed on the Connectivity Map (CMap) database [52]. First, COPD differentially expressed genes were organized as up-regulated and down-regulated gene lists. Next, the two lists were uploaded to CMap query website (https://clue.io/query) as COPD disease signature. The L1000 gene expression Touchstone dataset, which provides the transcriptomic profiles of genetically modified cell lines, was chosen as the reference in the analysis. From the initial analysis result on disease connectivity, we filtered out the results from the genetically modified cell lines undergone knockout or overexpression on the 52 candidate genes. Specifically, we examined the genes with the connectivity scores greater than 90% or smaller than -90% in the lung cancer cell line A549 and HCC515. The other seven cell lines in the reference Touchstone dataset were not included in the analysis as the cell lines are not derived from the lung tissue.

3.16 R for data analysis

All the data analysis, including data cleaning, pre-processing, integration, statistical test, and visualization, was implemented with R version 3.5.1 (2018-07-02). The coding was saved as R scripts for reproducibility (https://github.com/fwbioinfo/COPD_repurpose). The detailed R version information and the running environment are listed below:

```
> sessionInfo()

R version 4.2.2 (2022-10-31 ucrt)
Platform: x86_64-w64-mingw32/x64 (64-bit)
Running under: Windows 10 x64 (build 19045)

Matrix products: default

locale:
[1] LC_COLLATE=English_United States.1252  LC_CTYPE=English_United States.1252
[3] LC_MONETARY=English_United States.1252 LC_NUMERIC=C
[5] LC_TIME=English_United States.1252

attached base packages:
[1] grid      stats4    stats     graphics  grDevices utils     datasets  methods
[9] base

other attached packages:
 [1] RColorBrewer_1.1-3    fgsea_1.24.0          easyPubMed_2.13
 [4] igraph_1.5.0          enrichplot_1.18.4     clusterProfiler_4.6.2
 [7] conflicted_1.2.0      ggvenn_0.1.10         ggpubr_0.6.0
[10] ggplot2_3.4.1         annotate_1.76.0       XML_3.99-0.14
[13] hgug4112a.db_3.2.3    org.Hs.eg.db_3.16.0   AnnotationDbi_1.60.2
[16] IRanges_2.32.0        S4Vectors_0.36.2      limma_3.54.1
```

```
[19] dplyr_1.1.0           GEOquery_2.66.0      Biobase_2.58.0
[22] BiocGenerics_0.44.0   magrittr_2.0.3       tidyr_1.3.0
[25] data.table_1.14.8     DOSE_3.24.2

loaded via a namespace (and not attached):
  [1] ggtree_3.6.2           colorspace_2.1-0       gson_0.1.0
  [4] ggsignif_0.6.4         ellipsis_0.3.2         qvalue_2.30.0
  [7] XVector_0.38.0         aplot_0.1.10           farver_2.1.1
 [10] graphlayouts_1.0.0     ggrepel_0.9.3          bit64_4.0.5
 [13] scatterpie_0.2.1       fansi_1.0.4            xml2_1.3.3
 [16] codetools_0.2-18       splines_4.2.2          cachem_1.0.8
 [19] GOSemSim_2.24.0        polyclip_1.10-4        jsonlite_1.8.5
 [22] broom_1.0.5            GO.db_3.16.0           png_0.1-8
 [25] ggforce_0.4.1          readr_2.1.4            compiler_4.2.2
 [28] httr_1.4.6             reporttools_1.1.3      backports_1.4.1
 [31] lazyeval_0.2.2         Matrix_1.5-1           fastmap_1.1.1
 [34] cli_3.6.0              tweenr_2.0.2           tools_4.2.2
 [37] gtable_0.3.1           glue_1.6.2             GenomeInfoDbData_1.2.9
 [40] reshape2_1.4.4         tinytex_0.45           fastmatch_1.1-3
 [43] Rcpp_1.0.10            carData_3.0-5          cellranger_1.1.0
 [46] vctrs_0.5.2            Biostrings_2.66.0      nlme_3.1-160
 [49] ape_5.7-1              ggraph_2.1.0           xfun_0.39
 [52] stringr_1.5.0          openxlsx_4.2.5.2       lifecycle_1.0.3
 [55] rstatix_0.7.2          zlibbioc_1.44.0        MASS_7.3-58.1
 [58] scales_1.2.1           tidygraph_1.2.3        hms_1.1.2
 [61] parallel_4.2.2         memoise_2.0.1          gridExtra_2.3
 [64] ggfun_0.1.1            downloader_0.4         HDO.db_0.99.1
 [67] yulab.utils_0.0.6      stringi_1.7.12         RSQLite_2.3.1
 [70] tidytree_0.4.2         zip_2.3.0              BiocParallel_1.32.6
 [73] GenomeInfoDb_1.34.9    rlang_1.0.6            pkgconfig_2.0.3
 [76] bitops_1.0-7           lattice_0.20-45        purrr_1.0.1
 [79] treeio_1.22.0          patchwork_1.1.2        shadowtext_0.1.2
 [82] cowplot_1.1.1          bit_4.0.5              tidyselect_1.2.0
```

```
 [85] plyr_1.8.8           R6_2.5.1             generics_0.1.3
 [88] DBI_1.1.3            pillar_1.8.1         withr_2.5.0
 [91] KEGGREST_1.38.0      abind_1.4-5          RCurl_1.98-1.12
 [94] tibble_3.1.8         crayon_1.5.2         car_3.1-2
 [97] utf8_1.2.3           tzdb_0.3.0           viridis_0.6.3
[100] readxl_1.4.3         blob_1.2.4           digest_0.6.31
[103] xtable_1.8-4         gridGraphics_0.5-1   munsell_0.5.0
[106] ggplotify_0.1.1      viridisLite_0.4.1
```
